# Distinct Markers of Discordant Treatment Response to Lifestyle Intervention in MASLD, Independent of Weight Loss

**DOI:** 10.3390/biomedicines13092161

**Published:** 2025-09-04

**Authors:** Ling Luo, Congxiang Shao, Zhi Dong, Shuyu Zhuo, Shiting Feng, Wei Wang, Junzhao Ye, Bihui Zhong

**Affiliations:** 1 Department of Gastroenterology, The First Affiliated Hospital of Sun Yat-sen University, No. 58 Zhongshan II Road, Yuexiu District, Guangzhou 510080, China; luol27@mail2.sysu.edu.cn (L.L.); shaocx@mail2.sysu.edu.cn (C.S.); 2 Department of Radiology, The First Affiliated Hospital of Sun Yat-sen University, No. 58 Zhongshan II Road, Yuexiu District, Guangzhou 510080, China; dongzh7@mail.sysu.edu.cn (Z.D.); fengsht@mail.sysu.edu.cn (S.F.); 3 Department of Nutrition, The First Affiliated Hospital of Sun Yat-sen University, No. 58 Zhongshan II Road, Yuexiu District, Guangzhou 510080, China; zhuoshy@mail.sysu.edu.cn; 4 Department of Ultrasound, The First Affiliated Hospital of Sun Yat-sen University, No. 58 Zhongshan II Road, Yuexiu District, Guangzhou 510080, China; wangw73@mail.sysu.edu.cn

**Keywords:** metabolic dysfunction-associated steatotic liver disease, treatment response, weight loss, lifestyle intervention

## Abstract

**Background/Objectives:** Weight loss is the primary therapy for metabolic dysfunction-associated steatotic liver disease (MASLD). However, the proportion and factors influencing therapeutic changes in the liver condition contrary to weight loss remain unclear. **Methods**: This observational cohort study spanned between January 2015 and January 2024, with a 48-week lifestyle modification until January 2025. The liver fat content (LFC) determined using MRI-PDFF and liver stiffness measurement (LSM) via 2D-SWE were assessed at baseline and 48 weeks. The weight loss target (WLT) was determined as a reduction of ≥3% in body weight for lean/normal-weight patients and ≥5% for patients who were overweight/obese. **Results**: Overall, 397 patients with MASLD (30.5% achieving WLT) were included. For participants with WLT, 24.8% presented MRI-PDFF non-response, which was associated with moderate–vigorous physical activity (MVPA) ≥ 150 min/week, indicating a lower likelihood of non-response. Alanine aminotransferase (ALT) non-response occurred in 29.6% of patients and was linked to changes in LFC (ΔLFC, calculated as the baseline minus week 48). LSM non-response was observed in 48.2%, with high free fatty acid (FFA) levels identified as a risk factor. Among individuals without WLT, 29.0% demonstrated an MRI-PDFF response that correlated with greater reductions in low-density lipoprotein cholesterol; 39.4% exhibited an ALT response, which was associated with more significant reductions in LFC. The LSM response was 37.8%, also correlating with a reduction in LFC. **Conclusions**: Our results identified that MVPA, baseline steatosis degree, FFA, and their responses served as significant markers for treatment response contrary to weight loss in MASLD.

## 1. Introduction

Metabolic dysfunction-associated steatotic liver disease (MASLD) is the leading cause of chronic liver disease, affecting approximately 38% of the global population [1]. MASLD encompasses a spectrum of progressive conditions ranging from simple hepatic steatosis to metabolic dysfunction-associated steatohepatitis (MASH), cirrhosis, and hepatocellular carcinoma. This condition is associated with an elevated risk of poor liver prognosis, as well as cardiovascular and metabolic events, resulting in a substantial healthcare and socioeconomic burden on society [2]. Although the Food and Drug Administration has recently approved resmetirom for MASLD, histologic remission was observed in approximately one-quarter of patients [3]. Lifestyle interventions, including calorie-restricted diets and regular physical activity, remain the core effective regimens in the management of MASLD.

The clinical consensus across regions and liver diseases is the recommendation of weight management strategies through comprehensive lifestyle modifications as the first-line treatment for MASLD [4,5,6]. The weight loss target (WLT) was set as a ≥3% weight loss for lean/normal-weight individuals and ≥5% for non-lean individuals, which is crucial for substantial improvements in histologic features like steatosis, hepatocyte ballooning, inflammation, and fibrosis [7,8]. A meta-analysis of 43 intervention studies involving 2809 patients with MASLD summarized that a dose–response relationship existed between weight change and improvements in biochemical markers, as well as intrahepatic fat content or even liver histology [9]. Every 1.0 kg of weight loss significantly correlated with a 0.83-unit (95% confidence interval [CI]: 0.53 to 1.14, *p* < 0.001) reduction in alanine aminotransferase (ALT) (IU/L) and a 0.77 percentage point (95%CI: 0.51 to 1.03, *p* < 0.001) reduction in hepatic steatosis measured by histology or magnetic resonance imaging (MRI) [9]. However, achieving WLT through lifestyle changes could be maintained in only 30.0–32.3% of patients with MASLD [10,11]. A lack of consistency was observed in achieving the WLT and improvements in MASLD characteristics. Approximately 8% of patients with WLT exhibited aggravating hepatic fibrosis; conversely, 35% and 16% of patients without WLT displayed a significant reduction in hepatic steatosis and fibrosis, respectively [10,12]. These inconsistencies can reduce adherence to continuous lifestyle modifications and pose a major challenge for clinicians in the management of MASLD. Importantly, a gap exists in knowledge regarding the factors that contribute to the inconsistent effects of successful weight loss on the liver condition.

MRI-based proton density fat fraction (MRI-PDFF) is a reliable and accurate method for quantifying liver fat content (LFC), outperforming liver histology in monitoring longitudinal changes in patients with MASLD. A ≥ 30% reduction in LFC correlates with a higher likelihood of histological improvement [13]. Furthermore, improvements in fibrosis (≥1 stage) are strongly associated with the resolution of MASH. Two-dimensional shear wave elastography (2D-SWE), which has an area under the curve of 0.95 for detecting advanced fibrosis, offers a non-invasive alternative [14]. Therefore, using a well-characterized prospective cohort of MASLD patients monitored with MRI-PDFF and 2D-SWE in China, this study aimed to explore the association between achieving WLT and treatment response in hepatic steatosis, injury, and fibrosis and to identify potential influencing factors that provide novel insights into lifestyle strategies for MASLD treatment.

## 2. Materials and Methods

### 2.1. Study Design and Participants

This prospective, single-center, observational cohort study was conducted at the Fatty Liver Disease Center of the First Affiliated Hospital of Sun Yat-sen University (Guangzhou, China). The study was approved by the Ethics Committee of the First Affiliated Hospital, Sun Yat-sen university {[2014]112}. The study protocol was registered in 2014 with the definition of NAFLD, and the collected variables encompassed all indicators necessary for diagnosing MASLD. In the final analysis, the patients included in this study were chosen according to the most recent diagnostic criteria for MASLD, which includes the presence of hepatic steatosis and at least one cardiometabolic risk factor [6]. All study participants provided signed informed consent.

Between January 2015 and January 2024, consecutive patients with MASLD were screened and underwent a comprehensive baseline survey comprising a standard proforma questionnaire, physical examination, biochemical testing, and imaging assessment. In this study, diabetes mellitus was excluded as a potential confounding factor. Weight loss is a primary clinical manifestation of diabetes mellitus, and the administration of hypoglycemic medications (such as a peptide-1 receptor agonist) may interfere with monitoring changes in weight over time [15]. Individuals with metabolic dysfunction and alcohol-associated liver disease were excluded from participation. Additionally, patients meeting any of the following criteria were excluded: incomplete records, age < 18 years, decompensated cirrhosis, significant alcohol consumption (≥30 g/day for males and ≥20 g/day for females), presence of other liver diseases (e.g., hepatitis B, hepatitis C, autoimmune hepatitis, cholestatic or vascular liver disease, and drug-induced liver injury), severe systemic illnesses, malignancy, pregnancy, and breastfeeding.

### 2.2. Clinical and Metabolic Evaluation

Demographic data, physical activity, dietary intake, medical history, smoking status, and alcohol consumption were collected using standard proforma questionnaires (Supplementary File S1). Trained physicians (L.L. and C.-X.S.) evaluated anthropometric parameters including height, weight, waist circumference (WC), hip circumference, and blood pressure. Body mass index (BMI, kg/m^2^) was calculated by dividing weight (kg) by the square of height (m). The waist-to-hip ratio was defined as the ratio of WC to hip circumference. Hypertension was defined as having a blood pressure measurement ≥ 140/90 mmHg or the use of antihypertensive medications [16].

After overnight fasting for at least 8 h, blood specimens were collected and analyzed. Liver biochemistry, blood lipid profiles, free fatty acids (FFAs), fasting glucose (FBG), insulin (FINS), and uric acid (UA) levels were measured using an Abbott c8000 automatic biochemistry analyzer (Abbott, Abbott Park, IL, USA). Serum total bile acid levels were measured by the enzyme circulation method (AU5800, Beckman Coulter, Brea, CA, USA). Homeostasis model assessment of insulin resistance (HOMA-IR) index was calculated as

HOMA-IR = (FBG (mmol/L) × FINS (µU/mL))/22.5(1)

High FFA levels were determined as serum FFA ≥ 769 μmol/L [17]. The cutoff point for ALT levels was defined as 33 IU/L for men and 25 IU/L for women, while the cutoff point for γ-glutamyl transpeptidase (GGT) levels was set to 50 IU/L [6,18].

### 2.3. Hepatic Steatosis Assessment with MRI-PDFF

MRI-PDFF was performed on each participant using a 3.0-Tesla MRI scanner (Siemens 3.0 T Magnetom Verio; Siemens, Munchen, Germany) to assess LFC. The scanning protocol and imaging parameters for MRI-PDFF were previously described in our studies and are summarized as follows: TE1 2.5 ms, TE2 3.7 ms, repetition time of 5.47 ms, flip angle of 5°, ±504.0 kHz per pixel receiver bandwidth, and a slice thickness of 3.0 mm [19,20]. Hepatic steatosis is defined as an LFC of ≥5.0%, while moderate-to-severe steatosis is characterized by an LFC of ≥11.0% [6].

### 2.4. Liver Stiffness Measurements with 2D-SWE

All participants underwent 2D-SWE (Aix-en-Provence, France) to obtain liver stiffness measurements (LSM). After fasting for a minimum of 6 h, the patient was positioned in a supine position with the right arm fully extended to the side. The probe was placed in the right intercostal space. A rectangular region of interest (approximately 4 × 3 × 3 cm) located 1–2 cm below the liver capsule was selected, and measurements were conducted in a 2.0 cm diameter circular region of interest without focal lesions, biliary tracts, or blood vessels. The average of five consecutive measurements was calculated for each participant. For the evaluation of liver fibrosis, the following cutoff values were used: F0, ≤6.3 kpa; F1, 6.4–7.5 kpa; F2, 7.6–8.8 kpa; F3, 8.9–9.8 kpa; and F4, ≥9.9 kpa [14].

### 2.5. Lifestyle Intervention and Follow-Up

During the initial face-to-face visit, all patients with MASLD received lifestyle modification suggestions from a nutrition specialist (S.-Y.Z.) following a structured protocol based on current MASLD guidelines [4,5,6]. The counseling session lasted approximately 45 min and included personalized dietary plans and practical strategies to increase physical activity. The daily caloric requirements for each participant were estimated based on their body weight and physical activity levels. Subsequently, participants were provided with nutritional guidance through an easy-to-carry brochure that outlined the dietary caloric restriction menu (Figure S1). The calorie restriction program aimed for a reduction in daily energy intake from a baseline of at least 500 kcal, with a macronutrient distribution of 30% fat, 15% protein, and 55% carbohydrates [8]. Participants were instructed to consume 25 g of fiber and less than 250 mg of cholesterol daily. Moreover, participants were encouraged to engage in any kind of moderate–vigorous physical activity (MVPA), encompassing both recreational and occupational physical activities that induce perspiration, such as football, volleyball, bicycling, carrying or lifting heavy loads, and construction work, for at least 150 min per week [21]. During the follow-up, each participant was instructed to adhere to their personalized dietary and physical activity routines. They underwent clinical evaluations and lifestyle guideline assessments every 12 weeks until the 48-week visit. At each 12-week visit, follow-up interviews were conducted by the professional nutritionist (S.-Y.Z.). Participants were asked to report the frequency of physical activity and the quantity of their usual food intake during the most recent month, including meat, seafood, eggs, vegetables, fruit, and nuts (Supplementary File S1). The average daily energy intake was estimated based on the Chinese Food Composition Tables [22,23]. The changes in indicators during the observation period were calculated using the definitions of absolute change (Δ) and relative change (Δ%), which are defined as follows:

Δweight = baseline weight − week-48 weight(2)

Δ% weight = (baseline weight − week-48 weight)/baseline weight × 100%(3)

Furthermore, WLT success was defined as a weight loss (Δ% weight) of ≥3% for patients of a lean/normal weight (baseline BMI ≤ 23.0 kg/m^2^) and ≥5% for those who were overweight or obese [7,8]. For patients with indications for drug therapy, the supervising physician (B.-H.Z.) prescribed medications based on clinical guidelines [24,25]. The pharmacological therapy included statins for low-density lipoprotein (LDL) cholesterol control, fenofibrate for triglyceride (TG) control, and benzbromarone for UA control.

### 2.6. Study Endpoints

The study outcomes included the treatment response to liver steatosis, injury, and fibrosis at 48 weeks. For hepatic steatosis, an MRI-PDFF response was defined as a relative decrease in LFC values of ≥30% from baseline [6,13]. For liver injury, patients with elevated ALT at baseline were included in the analysis, and an ALT response was considered as an absolute decrease in ≥17 IU/L from baseline, according to the 2024 EASL-EASD-EASO Clinical Practice Guidelines [6]. For liver fibrosis, the analysis included individuals with a baseline fibrosis stage ≥1, defining an LSM response as a decline of ≥1 stage from baseline [e.g., a reduction from F2 (7.6–8.8 kpa) to F1 (6.4–7.5 kpa) or from F1 to F0 (≤6.3 kpa)].

### 2.7. Statistical Analysis

Continuous data were reported as the mean ± standard deviation or median (interquartile range) and compared using either an independent samples t-test or Mann–Whitney U test, depending on the data distribution. Categorical data were presented as the percentages and compared using the chi-square test. Univariable and multivariable logistic regression analyses were conducted for factors associated with the treatment response to liver steatosis, injury, or fibrosis. Demographic and anthropometric data, lifestyle habits, biochemical parameters, radiographic assessments, and changes in these variables were used for univariable logistic regression analysis. Given the exploratory nature of the univariable analyses employed to select candidate variables for the multivariable models, no correction for multiple comparisons was applied to minimize the risk of Type II error. Variables with *p* < 0.05 in the univariable analysis and potential confounders, such as age, sex, lipid-lowering drugs, and uric acid-lowering drugs, were included in the multivariable adjustments. Statistical analyses were performed using R language version 4.3.1. Two-tailed *p* values < 0.05 were considered statistically significant.

## 3. Results

### 3.1. Study Cohort Characteristics at Baseline and During Follow-Up

**Figure 1:**
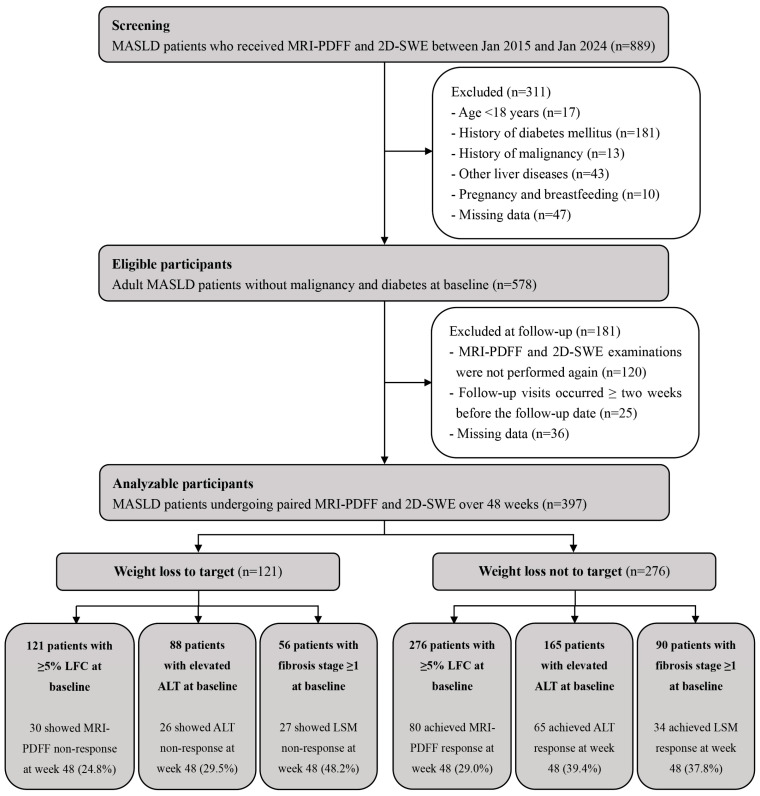
Flow diagram of participant recruitment, screening, and follow-up. Abbreviations: MASLD, metabolic dysfunction-associated steatotic liver disease; MRI-PDFF, magnetic resonance imaging-based proton density fat fraction; 2D-SWE, two-dimensional shear wave elastography; LFC, liver fat content; ALT, alanine aminotransferase; and LSM, liver stiffness measurement.

A total of 889 patients with MASLD who underwent concurrent MRI-PDFF and 2D-SWE were recruited (Figure 1). We excluded patients younger than 18 years (n = 17), those with diabetes mellitus (n = 181), malignancies (n = 13), other liver diseases (n = 43), pregnant or breastfeeding individuals (n = 10), those who did not undergo a second clinical assessment at 48 weeks (n = 120), those who did not follow-up on time (n = 25), and those with missing data (n = 83). Ultimately, this study included 397 consecutive patients with MASLD (mean age 42.4 ± 13.6 years; 71.0% male; BMI 26.8 ± 3.4 kg/m^2^; and 87.2% classified as overweight or obese), all of whom underwent paired evaluations with MRI-PDFF and 2D-SWE assessments at the baseline and 48 weeks. Clinical characteristics at the baseline and follow-up for the entire cohort are presented in Table 1. After 48 weeks of follow-up, significant differences were observed in the levels of weight, BMI, WC, diastolic blood pressure, serum lipids (except for high-density lipoprotein cholesterol), FINS, HOMA-IR, liver enzymes, and LFC (all *p* < 0.05, Table 1). Similar findings were observed in MASLD patients who achieved WLT (Table S1).

As detailed in Table 2, 46.9% of patients with MASLD achieved an energy reduction of ≥500 kcal/d from the baseline. Moreover, 63.2% consistently engaged in MVPA for at least 150 min per week, 32.5% received lipid-lowering drugs, and 9.8% received UA-lowering drugs. At 48 weeks, 121 (30.5%) patients with MASLD successfully achieved WLT. Participants with WLT tended to have poor baseline metabolic profiles and liver injury, including a high body weight, BMI, WC, LDL cholesterol, FFA, FINS, HOMA-IR, LFC, ALT, aspartate aminotransferase (AST), and LSM compared to those without WLT (all *p* < 0.05, Table 2). However, patients with WLT had a significantly higher proportion of participants achieving an energy intake reduction of ≥500 kcal/d compared to those without WLT (60.3% vs. 40.9%, *p* = 0.002, Table 2).

### 3.2. Clinical Characteristic Patterns of Treatment Response in Patients with MASLD

**Figure 2:**
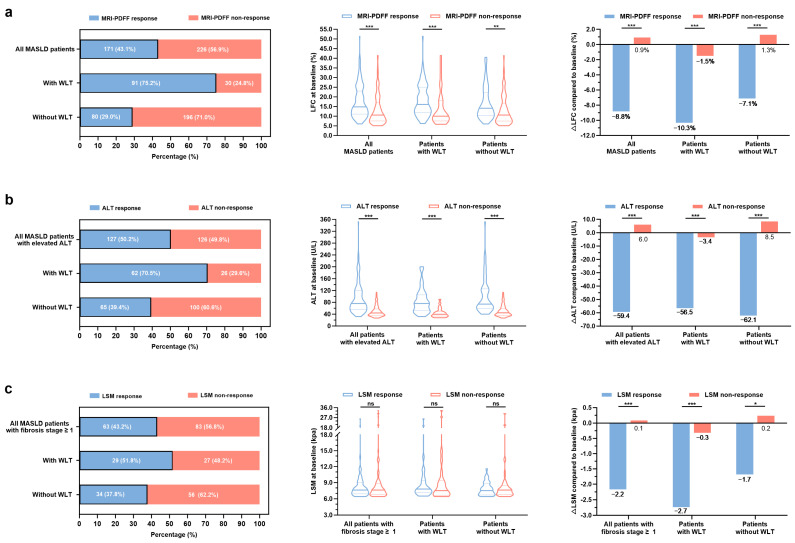
Characteristic clinical patterns of treatment response among all participants and those with or without weight loss to the target. **Percentage chart** showing the proportion of treatment responses to hepatic steatosis (**a**), hepatic injury (**b**), and hepatic fibrosis (**c**) at 48 weeks. **Violin plots** displaying the baseline LFC (**a**), ALT (**b**), or LSM (**c**) levels. **Bar graphs** displaying absolute changes from baseline in LFC (**a**), ALT (**b**), or LSM (**c**) levels. * *p* < 0.05, ** *p* < 0.01, *** *p* < 0.001, and ^ns^ *p*-non-significant. Abbreviations: MASLD, metabolic dysfunction-associated steatotic liver disease; WLT, weight loss target; MRI-PDFF, magnetic resonance imaging-based proton density fat fraction; LFC, liver fat content; ALT, alanine aminotransferase; LSM, liver stiffness measurement; and Δ, absolute change in indicators, i.e., baseline minus 48-week measurements.

At 48 weeks, 171 (43.1%) patients presented an MRI-PDFF response, with a significantly higher response rate in patients with WLT than in those without WLT (75.2% vs. 29.0%, *p* < 0.001) (Figure 2a). Compared with MRI-PDFF non-response patients, MASLD patients with an MRI-PDFF response had higher baseline LFC levels [10.6 (7.5, 17.0) vs. 14.9 (11.1, 23.0) %, *p* < 0.001], and subgroup analyses based on weight loss status demonstrated similar findings (all *p* < 0.01, Table S2). We further analyzed the treatment response to liver injury in 253 patients with elevated ALT levels at baseline and discovered that the ALT response rate was 50.2% in all patients, 70.5% in patients with WLT, and 39.4% in those without WLT (Figure 2b). Also, serum baseline ALT levels were significantly higher in participants with an ALT response than in those with no response (all *p* < 0.001, Table S3). For liver fibrosis, 146 patients with a baseline fibrosis stage ≥ 1 were included in the analysis, and LSM response rates were 43.2%, 51.8%, and 37.8% for all analyzable participants and for participants with or without WLT, respectively (Figure 2c). However, no significant difference was observed in baseline LSM values between patients with and without LSM responses (*p* > 0.05, Table S4). Additionally, large changes in LFC, ALT, and LSM values were observed in patients with treatment responses to liver steatosis, injury, and fibrosis (Figure 2). In the WLT group, patients with no response exhibited a trend of improvement after 48 weeks of treatment but did not reach clinical significance.

Furthermore, the correlation between treatment response in hepatic injury and fibrosis in 171 patients with an MRI-PDFF response and 226 patients with MRI-PDFF non-response is outlined in Figure S2. We observed that patients with an MRI-PDFF response demonstrated a higher proportion of ALT (48.5% vs. 19.5%, *p* < 0.001) and LSM responses (21.1% vs. 12.0%, *p* = 0.01) than those with MRI-PDFF non-response.

### 3.3. Factors Associated with Treatment Non-Response to Hepatic Steatosis, Injury, or Fibrosis in Patients with MASLD Undergoing WLT

**Figure 3:**
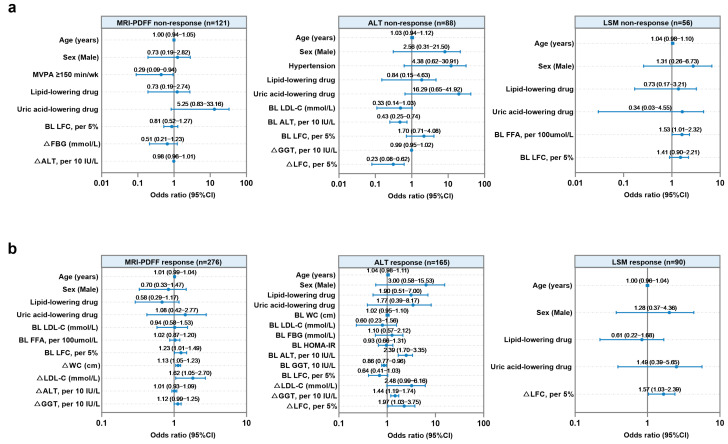
Factors associated with treatment responses to hepatic steatosis, injury, and fibrosis in patients with MASLD. (**a**) Forest plots illustrating multivariable analyses of factors associated with treatment non-responses to hepatic steatosis, injury, or fibrosis in patients with MASLD who achieved weight loss targets. (**b**) Forest plots illustrating multivariable analyses of factors associated with treatment responses to hepatic steatosis, injury, or fibrosis in patients with MASLD who did not achieve weight loss targets. Abbreviations: MVPA, moderate–vigorous physical activity; LFC, liver fat content; FBG, fasting glucose; ALT, alanine aminotransferase; LDL-C, low-density lipoprotein cholesterol; GGT, γ-glutamyl transpeptidase; FFA, free fatty acid; WC, waist circumference; HOMA-IR, homeostasis model assessment of insulin resistance; BL, baseline; and Δ, change in indicators, i.e., baseline minus 48-week measurements.

**Figure 4:**
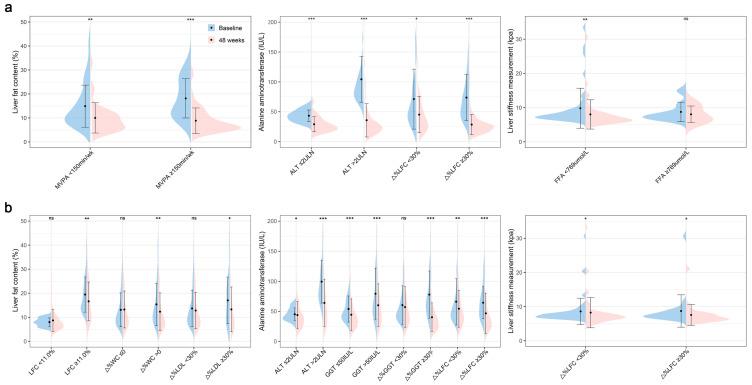
Changes in liver fat content, alanine aminotransferase, and liver stiffness during the 48-week follow-up period. (**a**) Liver characteristics subgrouped by the identified factors in patients who achieved weight loss targets. (**b**) Liver characteristics subgrouped by the identified factors in patients who did not achieve weight loss targets. * *p* < 0.05; ** *p* < 0.01; *** *p* < 0.001; and ^ns^ *p*-non-significant. Abbreviations: MVPA, moderate–vigorous physical activity; ALT, alanine aminotransferase; LFC, liver fat content; FFA, free fatty acid; WC, waist circumference; LDL, low-density lipoprotein cholesterol; GGT, γ-glutamyl transpeptidase; and Δ%, relative change in indicators, i.e., (baseline levels—48-week levels)/baseline levels × 100%.

The associations between treatment non-response and baseline characteristics, as well as changes in clinical and radiographic indicators, were examined in patients with MASLD who underwent WLT (Figure 3a, Table S5). Regarding treatment response to hepatic steatosis, the univariable analysis revealed that an MVPA ≥ 150 min/week, baseline LFC, reduction in FBG (ΔFBG), and reduction in ALT (ΔALT, per 10 IU/L decrease) were associated with MRI-PDFF non-response. After multivariable adjustments, MVPA ≥ 150 min/week (odds ratio [OR]: 0.29, 95% CI: 0.09–0.94, and *p* = 0.04) was associated with lower odds of treatment non-response to hepatic steatosis in patients with MASLD undergoing WLT (Figure 3a). Specifically, the subgroup with MVPA ≥ 150 min/week exhibited a significantly greater decrease in LFC at 48 weeks than the subgroup < 150 min/week (Figure 4a). For the hepatic injury treatment response, 88 patients with MASLD and elevated baseline ALT levels were included in univariable and multivariable analyses. Baseline ALT (per 10 IU/L increase) (OR: 0.43, 95% CI: 0.25–0.74, and *p* = 0.002) and a reduction in LFC (ΔLFC, per 5% increase) (OR: 0.23, 95% CI: 0.08–0.62, and *p* = 0.004) remained independently and significantly associated with ALT non-response (Figure 3a). This suggests that individuals with higher baseline ALT levels and greater reductions in LFC are more likely to exhibit an ALT response. A pronounced improvement in ALT levels was observed in the subgroup with high baseline ALT levels or an MRI-PDFF response (Δ% LFC ≥ 30%) (Figure 4a). Among 56 patients with a baseline fibrosis stage ≥1, univariable analysis demonstrated that FFA (per 100 μmol/L increase) and LFC (per 5% increase) at baseline were associated with LSM non-response. Furthermore, a multivariable analysis revealed that baseline FFA (per 100 μmol/L increase) (OR: 1.53, 95% CI: 1.01–2.32, and *p* = 0.048) remained a significant factor for the presence of treatment non-response to hepatic fibrosis. At 48 weeks, a significant decrease in LSM was observed in the subgroup with baseline FFA levels < 769 μmol/L (*p* < 0.001) but not in the subgroup with baseline FFA levels ≥ 769 μmol/L (*p* = 0.37) (Figure 4a).

### 3.4. Factors Associated with Treatment Response to Hepatic Steatosis, Injury, or Fibrosis in Patients with MASLD Without WLT

The factors associated with treatment response in patients with MASLD who did not achieve WLT are displayed in Figure 3b and Table S6. Among 276 patients without WLT, baseline LDL cholesterol, FFA, and LFC levels, as well as changes in WC (ΔWC), LDL cholesterol (ΔLDL), ALT (per 10 IU/L increase), and GGT (per 10 IU/L increase) were associated with an MRI-PDFF response. Further multivariable models revealed that elevated baseline LFC levels (OR: 1.23, 95% CI: 1.01–1.49, and *p* = 0.04), and greater reductions in WC (i.e., a higher positive value for ΔWC) (OR: 1.13, 95% CI: 1.05–1.23, and *p* = 0.002) and LDL cholesterol (i.e., a higher positive value for ΔLDL) (OR: 1.68, 95% CI: 1.05–2.70, and *p* = 0.03) were associated with an increased probability of treatment response to hepatic steatosis. Despite a worse baseline condition, significant improvements in hepatic steatosis were observed at 48 weeks in the subgroup with moderate-to-severe steatosis, decreased WC, or decreased LDL cholesterol (Figure 4b). Regarding the hepatic injury efficacy, 165 patients with elevated baseline ALT levels were included in this analysis. A multivariable analysis revealed that baseline ALT (per 10 IU/L increase) (OR: 2.39, 95% CI: 1.70–3.35, and *p* < 0.001), baseline GGT (per 10 IU/L increase) (OR: 0.86, 95% CI: 0.77–0.96, and *p* = 0.009), reduction in GGT (ΔGGT, per 10 IU/L increase) (OR: 1.44, 95% CI: 1.19–1.74, and *p* < 0.001), and reduction in LFC (ΔLFC, per 5% increase) (OR: 1.97, 95% CI: 1.03–3.75, and *p* = 0.04) remained significant markers of the treatment response to hepatic injury in individuals without WLT. Notably, a higher baseline ALT and greater reductions in GGT and LFC were associated with an increased likelihood of ALT response in patients without WLT (Figure 3). Subgroup analyses grouped according to these identified factors revealed differences in the extent of the ALT reduction at 48 weeks (Figure 4b). In addition, 90 patients with MASLD and a baseline fibrosis stage ≥ 1 were included in the analysis of hepatic fibrosis efficacy, displaying that increasing LFC change (OR: 1.57, 95% CI: 1.03–2.39, and *p* = 0.04) was related to achieving an LSM response. At 48 weeks, patients with MASLD and a decreased LFC exhibited a significant decrease in LSM levels (*p* = 0.02, Figure 4b).

## 4. Discussion

This study demonstrated that approximately 24.8%, 29.6%, and 48.2% of patients who achieved their weight loss goals remained non-responsive to treatment for liver steatosis, injury, and fibrosis, respectively. The corresponding factors were the duration of MVPA, changes in LFC, and baseline FFA levels. In contrast, significant improvements in liver steatosis, injury, and fibrosis were observed in 29.0%, 39.4%, and 37.8% of the patients who did not achieve their weight loss goals, respectively. Decreasing WC, LDL cholesterol, GGT, and LFC were markers for a high probability of treatment response to hepatic steatosis, injury, and fibrosis. These heterogeneous associations among the different weight loss groups may benefit clinicians in managing and monitoring patients undergoing MASLD treatment.

Notably, only 30.5% of patients with MASLD achieved WLT after 48 weeks of treatment. Individuals with WLT exhibited more severe anthropometric and metabolic abnormalities and liver injury at baseline than those without WLT. Similar findings were observed when individuals were grouped according to treatment response to liver conditions. This phenomenon has been reported in other studies investigating the factors associated with successful weight loss or treatment response [10,11,26]. It may be attributed to individuals with a high BMI exhibiting elevated metabolic rates and energy expenditure, increasing the likelihood of achieving significant weight loss and experiencing liver benefits through modest lifestyle modifications [27,28]. The link between baseline liver enzymes or steatosis and treatment response could also be explained by their high baseline levels, facilitating quantitatively large and thus measurable changes in these parameters during treatment [10].

In assessing the biological plausibility of the identified factors associated with the treatment response to hepatic steatosis in this study, it was interesting to note that MVPA was a primary protective factor in patients with MASLD and WLT. This suggests that a lack of MVPA might contribute to a poor treatment response, even in patients with MASLD who successfully lose weight using other approaches. Similarly, a recent meta-analysis involving 551 patients with MASLD demonstrated that, regardless of achieving WLT, 150 min of moderate-intensity physical activity per week was likely to result in a treatment response in MRI-measured liver fat (pooled OR: 3.51, 95% CI: 1.49 to 8.23) [29]. MVPA is crucial in MASLD management, the mechanism of which may involve changes in body composition, characterized by the loss of adipose tissue, gain of lean muscle mass, heightened cardiorespiratory fitness, and enhanced vascular biology through the reversal of endothelial dysfunction [30]. Substantial reductions in WC and LDL cholesterol have emerged as independent factors associated with an increased probability of treatment response to hepatic steatosis in patients with MASLD who did not achieve the WLT. This observation may be attributed to the fact that WC serves as a superior indicator of visceral adiposity to body weight [31]. Visceral adiposity is linked to hepatic insulin resistance and the severity of MASLD [32]. Furthermore, current evidence indicates multifactorial mechanisms for lipid-lowering agents in MASLD. Fibrates are known to reduce hepatic steatosis, macrophage infiltration, and inflammatory gene expression while enhancing the expression of genes involved in β-oxidation [33,34]. Beyond the cholesterol-lowering properties of statins, studies in animal models have shown that statins reduce the levels of pro-inflammatory cytokines, such as TNF-α, IL-1β, and IL-6, and modulate small guanine triphosphate-binding proteins, the proliferator-activated receptor α, and paraoxonase 1, thereby exerting anti-inflammatory and anti-fibrotic effects on MASLD [35]. Clinically, these findings imply that lipid-centric strategies, such as statins and PCSK-9 inhibitors, may provide benefits for patients with MASLD who did not achieve WLT, warranting validation in interventional studies.

Regarding the treatment response to liver injury, the improvement of hepatic steatosis was a critical factor associated with decreased liver enzyme levels in MASLD, irrespective of WLT status. A reduction in liver triglyceride accumulation is accompanied by enhancements in insulin resistance, inflammatory response, and oxidative stress within the liver, all of which are essential factors in the reversal of hepatic injury [36]. Regarding the efficacy in improving liver fibrosis, high baseline FFA levels were identified as an independent factor for non-response to treatment, even in the presence of successful weight loss. Circulating FFAs primarily originate from the hydrolysis of triacylglycerol and are associated with progressive liver inflammation and fibrosis. The potential mechanism linking serum FFAs with MASLD progression involves FFA-induced hepatocellular apoptosis and injury by activating the pro-apoptotic protein Bax in a c-Jun N-terminal kinase-dependent manner [37]. Increased systemic oxidative stress is a pathophysiological hallmark of MASLD [38]. Thus, another possible explanation for the elevated FFAs in fibrosis non-responders is that FFAs act as strong oxidants in several pathological states, such as metabolic syndrome and diabetes [39]. Our study suggests an association between FFA levels and MASLD fibrosis efficacy, offering a snapshot of the circulating FFA state. However, whether increased serum FFA levels are a cause or a consequence of poor treatment response in hepatic fibrosis needs to be determined in future studies.

This study had several limitations. First, a liver biopsy combined with histological scoring remains the gold standard for assessing the response to MASLD treatment. Nonetheless, our study employed MRI-PDFF and 2D-SWE, which are non-invasive and relatively accurate techniques. These methods are better suited for large-scale studies in the general population. Second, our study participants were recruited from tertiary care centers and may not fully represent the typical MASLD cases encountered in primary care settings. Third, the exclusion of patients with diabetes mellitus was necessary to mitigate the confounding effects of the disease and its medications (e.g., GLP-1 receptor agonists, SGLT2 inhibitors) on weight and metabolic parameters. However, this exclusion implies that our cohort does not represent a significant and clinically relevant segment of the MASLD population. The altered metabolic milieu in type 2 diabetes mellitus (T2DM), characterized by more severe insulin resistance, dyslipidemia, and potential pancreatic beta-cell dysfunction, could fundamentally influence the treatment response factors we identified. For instance, the roles of physical activity, lipids, and FFAs may differ in the presence of T2DM or its pharmacotherapies. Therefore, our findings cannot be extrapolated to patients with diabetes mellitus. This critical gap implies the necessity for future studies specifically designed with cohorts that include patients with T2DM to validate our findings, representing a crucial next step in this line of research. Fourth, recall bias may exist when patients complete the self-report questionnaire. Our results should be interpreted with caution, as the potential for variable adherence to lifestyle interventions, which may not be fully captured by our methods, could influence the classification of response and non-response. While MVPA was a key factor, the study did not collect data on the precise intensity, duration, or type of physical activity. Therefore, we were unable to conduct further subgroup analyses that could potentially impact the effects of physical activity beyond the binary classification of meeting the threshold of ≥150 min/week. Future studies that incorporate detailed activity information, such as qualitative data from nutritionist notes, will offer deeper insights into the dose–response relationship between physical activity and treatment response. Additionally, this study defined WLT success using clinically relevant thresholds (≥3% for lean patients and ≥5% for overweight/obese patients), which are associated with improvements in steatosis and liver enzymes [7,8]. However, some guidelines suggest that a greater weight loss (7–10%) is often required for a significant histological improvement, particularly for fibrosis regression [6]. It is plausible that implementing a more stringent definition of WLT (e.g., ≥7%) could alter the composition of the responder and non-responder groups, potentially changing the identified factors. For instance, some patients achieving a 5% weight loss might be reclassified as non-responders, while the “response without WLT” group may diminish. This highlights the context-dependent nature of our findings based on the selected WLT criteria and suggests a direction for further investigation. Finally, this is a single-center study in China and includes a relatively small sample size, which limits the robustness and generalizability of the findings, especially in the analysis of liver fibrosis efficacy. Therefore, further long-term observational studies involving large cohorts of patients with significant fibrosis are warranted.

## 5. Conclusions

In summary, this study suggests that approximately a quarter to half of the Chinese patients with MASLD exhibit inconsistent associations between successful weight loss and treatment response in terms of liver characteristics. Clinical parameters at baseline and changes during treatment helped identify the therapeutic response in patients with MASLD, and the factors associated with a treatment response for hepatic steatosis, injury, and fibrosis varied according to weight loss status. These findings may help develop targeted and clinically feasible MASLD management strategies.

## Figures and Tables

**Table 1:** Characteristics of the study population at baseline and follow-up.

Characteristics	Baseline (n = 397)	48 Weeks (n = 397)	Change	*p*
Body weight (kg)	74.6 ± 12.1	72.8 ± 11.9	1.8 ± 4.0	0.04
Body mass index (kg/m^2^)	26.8 ± 3.4	26.2 ± 3.4	0.6 ± 1.4	0.009
Waist circumference (cm)	90.0 ± 8.2	88.6 ± 7.8	1.5 ± 4.8	0.01
Waist-to-hip ratio	0.90 ± 0.05	0.90 ± 0.05	0.00 ± 0.04	0.46
Systolic blood pressure (mmHg)	131.8 ± 16.5	129.3 ± 17.0	2.5 ± 15.5	0.04
Diastolic blood pressure (mmHg)	86.1 ± 11.7	83.2 ± 11.4	2.9 ± 12.3	0.001
Total cholesterol (mmol/L)	5.06 ± 1.04	4.78 ± 0.98	0.28 ± 1.04	<0.001
Triglyceride (mmol/L)	1.55 (1.08, 2.16)	1.44 (0.99, 1.99)	0.09 (−0.26, 0.56)	0.02
HDL cholesterol (mmol/L)	1.17 ± 0.25	1.17 ± 0.29	0.00 ± 0.21	0.71
LDL cholesterol (mmol/L)	3.17 ± 0.78	2.96 ± 0.73	0.21 ± 0.79	<0.001
Free fatty acid (μmol/L)	517 (418, 643)	500 (400, 609)	6.0 (−108, 149)	0.09
Fasting glucose (mmol/L)	4.9 (4.5, 5.4)	4.9 (4.5, 5.4)	0.0 (−0.4, 0.4)	0.81
Fasting insulin (uU/mL)	10.1 (7.3, 14.7)	9.2 (6.7, 12.5)	1.1 (−1.2, 3.7)	0.002
HOMA-IR	2.34 (1.65, 3.46)	2.09 (1.39, 2.89)	0.29 (−0.39, 0.90)	0.003
Uric acid (μmol/L)	420.6 ± 103.1	413.1 ± 107.6	7.2 ± 102.4	0.32
Alanine aminotransferase (IU/L)	40.0 (25.0, 67.5)	30.0 (21.0, 45.0)	5.0 (−5.0, 25.0)	<0.001
Aspartate aminotransferase (IU/L)	31.0 (23.0, 42.5)	25.0 (21.0, 34.0)	3.0 (−2.0, 13.0)	<0.001
γ-glutamyl transpeptidase (IU/L)	41.0 (27.0, 62.0)	32.0 (22.0, 49.5)	4.0 (−3.0, 22.0)	<0.001
Alkaline phosphatase (IU/L)	76.0 (66.0, 86.0)	73.0 (64.0, 84.8)	2.0 (−4.0, 9.0)	0.04
Total bilirubin (μmol/L)	12.6 (9.9, 16.5)	12.8 (10.3, 16.5)	0.0 (−2.3, 2.0)	0.64
Albumin (g/L)	45.7 ± 3.1	45.4 ± 3.0	0.4 ± 2.8	0.09
Total bile acid (μmol/L)	2.8 (1.8, 3.9)	2.5 (1.6, 3.9)	0.1 (−0.8, 1.3)	0.04
Liver fat content (%)	12.4 (8.7, 20.0)	9.1 (6.1, 14.5)	2.9 (−0.4, 6.3)	<0.001
Liver stiffness measurement (kpa)	5.9 (5.1, 7.1)	5.8 (5.0, 6.7)	0.0 (−0.3, 0.8)	0.18

Continuous variables that are normally distributed are reported as mean ± standard deviation, while continuous variables that are not normally distributed are presented as the median (interquartile range). Abbreviations: HDL cholesterol, high-density lipoprotein cholesterol; LDL cholesterol, low-density lipoprotein cholesterol; and HOMA-IR, homeostasis model assessment of insulin resistance.

**Table 2:** Clinical baseline characteristics of patients with MASLD.

Characteristics	Total (n = 397)	With WLT (n = 121)	Without WLT (n = 276)	*p*
Age (years)	42.4 ± 13.6	42.9 ± 13.7	42.1 ± 13.6	0.59
Male, n (%)	282 (71.0%)	79 (65.3%)	203 (73.6%)	0.10
Smoking, n (%)	44 (11.1%)	12 (9.9%)	32 (11.6%)	0.62
Body weight (kg)	74.6 ± 12.1	76.6 ± 12.5	73.7 ± 11.9	0.03
Body mass index (kg/m^2^)	26.8 ± 3.4	27.6 ± 3.5	26.5 ± 3.4	0.005
Waist circumference (cm)	90.0 ± 8.2	91.4 ± 8.7	89.4 ± 7.9	0.03
Waist-to-hip ratio	0.90 ± 0.05	0.90 ± 0.05	0.90 ± 0.05	0.37
Systolic blood pressure (mmHg)	131.8 ± 16.5	131.0 ± 16.2	132.2 ± 16.6	0.50
Diastolic blood pressure (mmHg)	86.1 ± 11.7	86.4 ± 11.8	86.0 ± 11.7	0.74
Total cholesterol (mmol/L)	5.06 ± 1.04	5.21 ± 1.13	5.00 ± 1.00	0.07
Triglyceride (mmol/L)	1.55 (1.08, 2.16)	1.54 (1.02, 2.14)	1.58 (1.12, 2.19)	0.47
HDL cholesterol (mmol/L)	1.17 ± 0.25	1.17 ± 0.22	1.17 ± 0.27	0.95
LDL cholesterol (mmol/L)	3.17 ± 0.78	3.32 ± 0.85	3.10 ± 0.74	0.02
Free fatty acid (μmol/L)	517 (418, 643)	563 (461, 690)	501 (412, 616)	0.02
Fasting glucose (mmol/L)	4.9 (4.5, 5.4)	4.9 (4.5, 5.5)	4.9 (4.5, 5.3)	0.73
Fasting insulin (uU/mL)	10.1 (7.3, 14.7)	11.1 (7.8, 16.5)	10.0 (7.2, 13.9)	0.04
HOMA-IR	2.34 (1.65, 3.46)	2.51 (1.79, 3.80)	2.22 (1.55, 3.32)	0.03
Uric acid (μmol/L)	420.6 ± 103.1	421.6 ± 106.8	420.1 ± 101.6	0.90
Alanine aminotransferase (IU/L)	40.0 (25.0, 67.5)	46.0 (29.5, 78.5)	37.0 (23.0, 62.8)	0.01
Aspartate aminotransferase (IU/L)	31.0 (23.0, 42.5)	34.0 (24.5, 48.5)	29.5 (22.0, 40.0)	0.008
γ-glutamyl transpeptidase (IU/L)	41.0 (27.0, 62.0)	45.0 (28.0, 66.5)	39.0 (26.0, 60.8)	0.17
Alkaline phosphatase (IU/L)	76.0 (66.0, 86.0)	77.0 (68.0, 87.0)	76.0 (64.0, 86.3)	0.22
Total bilirubin (μmol/L)	12.6 (9.9, 16.5)	13.2 (10.3, 16.6)	12.3 (9.7, 16.4)	0.18
Albumin (g/L)	45.7 ± 3.1	45.5 ± 3.1	45.8 ± 3.1	0.34
Total bile acid (μmol/L)	2.8 (1.8, 3.9)	2.7 (2.0, 3.8)	2.9 (1.8, 3.9)	0.60
Liver fat content (%)	12.4 (8.7, 20.0)	14.9 (10.2, 24.1)	11.4 (8.1, 18.2)	<0.001
Liver stiffness measurement (kpa)	5.9 (5.1, 7.1)	6.2 (5.2, 7.7)	5.8 (5.0, 6.8)	0.01
Elevated ALT ^1^, n (%)	253 (63.7%)	88 (72.3%)	165 (59.8%)	0.01
Fibrosis ≥ stage 1, n (%)	146 (36.8%)	56 (46.3%)	90 (32.6%)	0.009
MVPA ≥ 150 min/wk, n (%)	251 (63.2%)	82 (67.8%)	169 (61.2%)	0.31
Reduced energy intake ≥ 500 kcal/d, n (%)	186 (46.9%)	73 (60.3%)	113 (40.9%)	0.002
Lipid-lowering drug, n (%)	129 (32.5%)	36 (29.8%)	93 (33.7%)	0.44
Uric acid-lowering drug, n (%)	39 (9.8%)	11 (9.1%)	28 (10.1%)	0.75

Continuous variables that are normally distributed are reported as mean ± standard deviation, while continuous variables that are not normally distributed are presented as the median (interquartile range). Categorical data are expressed as frequency (percentage). Abbreviations: WLT, weight loss target; HDL cholesterol, high-density lipoprotein cholesterol; LDL cholesterol, low-density lipoprotein cholesterol; HOMA-IR, homeostasis model assessment of insulin resistance; ALT, alanine aminotransferase; and MVPA, moderate–vigorous physical activity. ^1^ Elevated ALT was defined as >33 U/L for men and >25 U/L for women.

## Data Availability

The original contributions presented in this study are included in the article. Further inquiries can be directed to the corresponding authors.

## Supplementary Materials

The following supporting information can be downloaded at: https://www.mdpi.com/article/10.3390/biomedicines13092161/s1, Figure S1: Dietary management; Figure S2: Sankey diagram illustrating the correlation between treatment response to liver injury as assessed by ALT and liver fibrosis as measured by LSM in MASLD patients with (A) or without (B) MRI-PDFF response; Table S1: Parameter changes at two points in patients with MASLD stratified by weight loss status; Table S2: Baseline characteristics of patients with MASLD stratified by MRI-PDFF response status; Table S3: Baseline characteristics of patients with MASLD stratified by ALT response status; Table S4: Baseline characteristics of patients with MASLD stratified by LSM response status; Table S5: Factors associated with treatment non-response to hepatic steatosis, injury, or fibrosis in patients with MASLD who achieved weight loss targets; Table S6: Factors associated with treatment response to hepatic steatosis, injury, or fibrosis in patients with MASLD who did not achieve weight loss targets; and Supplementary File S1.

## Abbreviations

The following abbreviations are used in this manuscript:

MASLD	Metabolic dysfunction-associated steatotic liver disease
MASH	Metabolic dysfunction-associated steatohepatitis
WLT	Weight loss target
CI	Confidence interval
ALT	Alanine aminotransferase
MRI	Magnetic resonance imaging
MRI-PDFF	Magnetic resonance imaging-based proton density fat fraction
LFC	Liver fat content
2D-SWE	Two-dimensional shear wave elastography
WC	Waist circumference
BMI	Body mass index
FFA	Free fatty acid
FBG	Fasting glucose
FINS	Fasting insulin
UA	Uric acid
HOMA-IR	Homeostasis model assessment of insulin resistance
GGT	γ-glutamyl transpeptidase
LSM	Liver stiffness measurements
MVPA	Moderate-vigorous physical activity
LDL	Low-density lipoprotein cholesterol
TG	Triglyceride
