# Supplementary material for: Distinct Markers of Discordant Treatment Response to Lifestyle Intervention in MASLD, Independent of Weight Loss
Source: Biomedicines. 2025 Sep 4;13(9):2161. doi: 10.3390/biomedicines13092161 (PMC12466973; doi:10.3390/biomedicines13092161)
Supplement: Supplementary file 1 [file biomedicines-13-02161-s001.zip › biomedicines-3761775-supplementary.pdf]

a An easy-to-carry brochure for managing fatty live disease

Patient Education Manual for Fatty Liver Disease

Diet and exercise

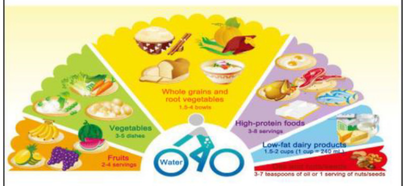

The Fatty Liver Clinic of the First Affiliated Hospital of Sun Yat-sen University

2014 (Third Edition)

Fatty Liver Health Promotion

—— Take Care of Your " 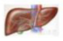 "

Consultation Hours: Thursdays, 3:00-5:00 PM

Consulting Specialist: Professor Zhong Bihui

Location: 5th floor, Building 2

Tel: (8620)87766335

31

Method for Calculating Daily Calories

Step 1: Ideal Body Weight

Calculation Formula: Ideal Body Weight (kg) = Height (cm) – 105

Step 2: Evaluate Current Weight Status (Percentage Above Ideal Weight)

Calculation Formula: (Actual Weight - Ideal Weight) / Ideal Weight * 100%

Actual Weight	≥40%	≥20%	≥10%	-10%~10%	≤-10%	≤-20%
Definition	Severe obesity	Obesity	Overweight	Normal weight	Underweight	Emaciated

Step 3: Calculate Total Daily Calories:

Total Daily Calories (kcal) = Ideal Weight × Caloric Intake per kg of Ideal Weight per day

Physical Activity Level	Caloric Intake Required (kcal/kg of Ideal Body Weight per day)		
	Underweight	Normal weight	Overweight / Obesity
Bedridden	20-25	15-20	15
Light Physical Activity	35	30	20-25
Moderate Physical Activity	40	35	30
Heavy Physical Activity	45-50	40	35

4

b Examples of dietary calorie restriction menus

1200 kcal recipe

Suitable for those who need 1200-1300 kcal daily. Use 15 grams of cooking oil (1.5 tablespoons) and 6 grams of salt (3 grams for hypertensive patients, 2 grams for hypertensive patients with diabetes) throughout the day.

Breakfast (Choose any one)	
1	Low-fat milk/soy milk (1 cup/250ml), boiled egg (1)<br>Soda cracker (5 pieces)
2	Low-fat milk/soy milk (1 cup/250ml), boiled egg (1)<br>Steamed bun (1), fresh tomato (1)
3	Low-fat milk/soy milk (1 cup/250ml), boiled egg (1)<br>Vegetable salad (1 small bowl), savory bread (2 pieces)
**Lunch (Choose any one)**	
1	Steamed rice (1 small bowl/100g)<br>Tir-fried shredded vegetables (100g green bell pepper, 100g jute stem, 50g pork)<br>Mushroom and tofu soup (50g mushrooms, 50g tofu)
2	Steamed rice (1 small bowl/100g)<br>Stir-fried bottle gourd with scrambled eggs (80g bottle gourd, 50g eggs)<br>Fish ball and cabbage soup (50g fish meat, 200g cabbage)
3	Noodles (1 small bowl/100g)

1500 kcal recipe:

Suitable for those who need 1500-1600 kcal daily. Use 15 grams of cooking oil. (1.5 tablespoons) and 6 grams of salt (3 grams for hypertensive patients, 2 grams for hypertensive patients with diabetes) .

Breakfast (Choose any one)	
1	Low-fat milk/soy milk (250ml), boiled egg (1), steamed twisted roll (1)
2	Low-fat milk/soy milk (250ml), boiled egg (1), steamed bun(1)
3	Low-fat milk/soy milk (250ml), boiled egg (1), Sugar-free rice cake (1)
**Lunch (Choose any one)**	
1	Rice (1 small bowl/100g)<br>Stir-fried pork slices with zucchini (50g lean pork, 100g zucchini)<br>Stir-fried baby bok choy with shiitake mushrooms (5g shiitake mushrooms, 150g baby bok choy)<br>Dried shrimp and seaweed soup (5g dried shrimp, 2g seaweed, 25g cucumber)
2	Rice (1 small bowl/100g)<br>Poached chicken (100g)<br>Spinach soup (50g spinach, 2g seaweed)
3	Pastry (1 sheet)<br>Kaiyang bean sprouts (Mung bean sprouts 100g, Kaiyang 5-6)<br>Meatballs (Lean pork 100g)<br>Tomato and egg soup (1 tomato, 1 egg)

1700 kcal recipe

Suitable for those who need 1700-1800 kcal daily. Use 25 grams of cooking oil. (2.5 tablespoons) and 6 grams of salt (3 grams for hypertensive patients, 2 grams for hypertensive patients with diabetes) throughout the day.

Breakfast (Choose any one)	
1	Low-fat milk/soy milk (250ml), boiled egg (1), steamed twisted roll (1)
2	Low-fat milk/soy milk (1 cup /250ml), steamed bun (1 piece), meat floss (1 small dish)
3	Low fat milk/soy milk (1 cup /250ml), boiled egg (1), salted bread (2 slices), mixed cucumber (half)
**Lunch (Choose any one)**	
1	Rice (1 bowl / 150g)<br>Seaweed salad (100g wet seaweed)<br>Sliced cauliflower (50g lean pork, 100g cauliflower)<br>Loofah Egg Soup (50g loofah, 50g egg)
2	Rice (1 bowl / 150g)<br>Spare ribs with kelp (100g spare ribs, 100g wet kelp)<br>Stir-fried cabbage with plain (cabbage 200g)
3	Scallion cake (standard flour 100g)<br>Stir-fried three shreds (lean pork 50g, green pepper 100g, water stem 100g)<br>Mixed radish and jellyfish (radish 100g, jellyfish 50g)<br>Prawn skin and seaweed soup (50g prawn skin, 2g seaweed)

Figure S1 Dietary management.

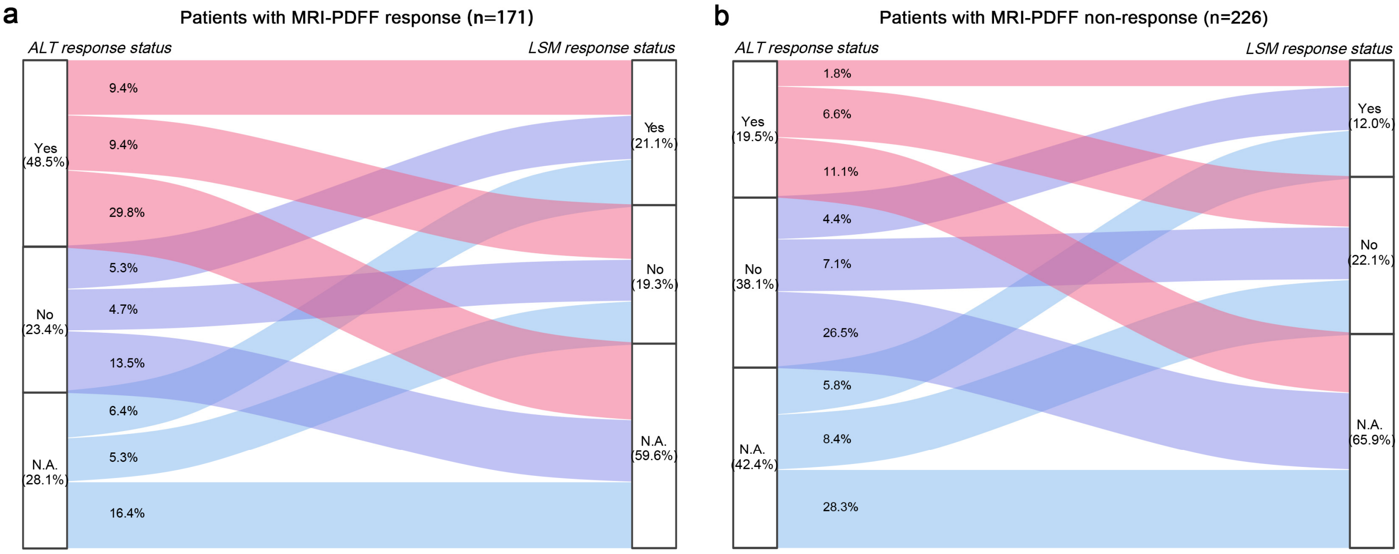

Figure S2 Sankey diagram illustrating the correlation between treatment response to liver injury as assessed by ALT and liver fibrosis as measured by LSM in MASLD patients with (A) or without (B) MRI-PDFF response.

Abbreviation: MASLD, metabolic dysfunction-associated steatotic liver disease; MRI-PDFF, magnetic resonance imaging-based proton density fat fraction; ALT, alanine aminotransferase; LSM, liver stiffness measurement.

N.A., not applicable. For liver injury efficacy analysis, patients with normal ALT at baseline were excluded. For liver fibrosis efficacy analysis, patients without liver fibrosis at baseline were excluded in the analysis.

Table S1. Parameter changes at two points in patients with MASLD stratified by weight loss status.

**Characteristics**	**With WLT (n=121)**			**Without WLT (n=276)**		
	**Baseline**	**48 weeks**	***P***	**Baseline**	**48 weeks**	***P***
Body weight (kg)	76.6 ± 12.5	70.2 ± 11.1	<0.001	73.7 ± 11.9	74.0 ± 12.1	0.80
Body mass index (kg/m^2)	27.6 ± 3.5	25.3 ± 3.0	<0.001	26.5 ± 3.4	26.6 ± 3.4	0.76
Waist circumference (cm)	91.4 ± 8.7	86.6 ± 7.3	<0.001	89.4 ± 7.9	89.4 ± 7.8	0.98
Waist-to-hip ratio	0.90 ± 0.05	0.89 ± 0.05	0.03	0.90 ± 0.05	0.90 ± 0.05	0.53
Systolic blood pressure (mmHg)	131.0 ± 16.2	128.0 ± 16.2	0.16	132.2 ± 16.6	129.9 ± 17.4	0.12
Diastolic blood pressure (mmHg)	86.4 ± 11.8	82.2 ± 11.0	0.005	86.0 ± 11.7	83.6 ± 11.6	0.02
Total cholesterol (mmol/L)	5.21 ± 1.13	4.62 ± 0.96	<0.001	5.00 ± 1.00	4.85 ± 0.98	0.07
Triglyceride (mmol/L)	1.54 (1.02, 2.14)	1.18 (0.91, 1.62)	0.002	1.58 (1.12, 2.19)	1.56 (1.04, 2.05)	0.48
HDL cholesterol (mmol/L)	1.17 ± 0.22	1.19 ± 0.26	0.47	1.17 ± 0.27	1.17 ± 0.30	0.98
LDL cholesterol (mmol/L)	3.32 ± 0.85	2.87 ± 0.70	<0.001	3.10 ± 0.74	2.99 ± 0.73	0.08
Free fatty acid (μmol/L)	563 (461, 690)	500 (397, 594)	0.004	501 (412, 616)	504 (406, 613)	0.90
Fasting glucose (mmol/L)	4.9 (4.5, 5.5)	4.8 (4.4, 5.2)	0.17	4.9 (4.5, 5.3)	4.9 (4.5, 5.5)	0.52
Fasting insulin (uU/mL)	11.1 (7.8, 16.5)	8.7 (6.1, 11.8)	<0.001	10.0 (7.2, 13.9)	9.7 (7.0, 13.2)	0.43
HOMA-IR	2.51 (1.79, 3.80)	1.87 (1.19, 2.65)	<0.001	2.22 (1.55, 3.32)	2.22 (1.55, 3.14)	0.65
Uric acid (μmol/L)	421.6 ± 106.8	403.5 ± 115.9	0.21	420.1 ± 101.6	417.3 ± 103.7	0.75
Alanine aminotransferase (IU/L)	46.0 (29.5, 78.5)	23.0 (17.0, 34.0)	<0.001	37.0 (23.0, 62.8)	35.0 (23.0, 52.0)	0.07
Aspartate aminotransferase (IU/L)	34.0 (24.5, 48.5)	23.0 (20.0, 27.0)	<0.001	29.5 (22.0, 40.0)	27.0 (22.0, 36.0)	0.04
γ-glutamyl transpeptidase (IU/L)	45.0 (28.0, 66.5)	25.0 (19.0, 34.0)	<0.001	39.0 (26.0, 60.8)	34.5 (25.0, 57.0)	0.25
Alkaline phosphatase (IU/L)	77.0 (68.0, 87.0)	73.0 (64.5, 85.5)	0.03	76.0 (64.0, 86.3)	74.0 (64.0, 84.0)	0.26
Total bilirubin (μmol/L)	13.2 (10.3, 16.6)	13.1 (10.3, 16.3)	0.83	12.3 (9.7, 16.4)	12.7 (10.3, 16.9)	0.52
Albumin (g/L)	45.5 ± 3.1	45.3 ± 3.4	0.55	45.8 ± 3.1	45.4 ± 2.9	0.10
Total bile acid (μmol/L)	2.7 (2.0, 3.8)	2.3 (1.6, 3.5)	0.09	2.9 (1.8, 3.9)	2.6 (1.7, 3.9)	0.16
Liver fat content (%)	14.9 (10.2, 24.1)	7.1 (5.6, 11.3)	<0.001	11.4 (8.1, 18.2)	10.2 (7.2, 17.6)	0.02
Liver stiffness measurement (kpa)	6.2 (5.2, 7.7)	5.9 (5.1, 7.1)	0.13	5.8 (5.0, 6.8)	5.7 (5.0, 6.7)	0.58

Continuous variables that are normally distributed are reported as mean ± standard deviation, while continuous variables that are not normally distributed are presented as median (interquartile range). Abbreviation: MASLD, metabolic dysfunction-associated steatotic liver disease; WLT, weight loss target; HDL cholesterol, high-density lipoprotein cholesterol; LDL cholesterol, low-density lipoprotein cholesterol; HOMA-IR, homeostasis model assessment of insulin resistance.

Table S2. Baseline characteristics of patients with MASLD stratified by MRI-PDFF response status.

**Characteristics**	**With WLT (n=121)**			**Without WLT (n=276)**		
	**MRI-PDFF response (n=91)**	**MRI-PDFF non-response (n=30)**	***P***	**MRI-PDFF response (n=80)**	**MRI-PDFF non-response (n=196)**	***P***
Age (years)	42.7 ± 13.8	43.7 ± 13.5	0.73	43.3 ± 13.4	41.7 ± 13.7	0.36
Male, n (%)	60 (65.9%)	19 (63.3%)	0.80	54 (67.5%)	149 (76.0%)	0.15
Smoking, n (%)	11 (12.1%)	1 (3.3%)	0.32	7 (8.8%)	25 (12.8%)	0.33
Body weight (kg)	75.6 ± 13.1	79.6 ± 10.0	0.13	72.7 ± 11.4	74.1 ± 12.1	0.38
Body mass index (kg/m^2)	27.3 ± 3.6	28.4 ± 2.8	0.12	26.6 ± 3.5	26.5 ± 3.3	0.72
Waist circumference (cm)	91.0 ± 9.4	92.6 ± 5.9	0.29	89.7 ± 8.0	89.3 ± 7.9	0.71
Waist-to-hip ratio	0.90 ± 0.05	0.90 ± 0.04	0.79	0.90 ± 0.04	0.90 ± 0.05	0.53
Systolic blood pressure (mmHg)	130.3 ± 16.7	133.1 ± 14.5	0.41	135.6 ± 16.5	130.7 ± 16.5	0.03
Diastolic blood pressure (mmHg)	86.7 ± 12.2	85.6 ± 10.8	0.65	88.8 ± 11.6	84.8 ± 11.5	0.01
Total cholesterol (mmol/L)	5.26 ± 1.13	5.05 ± 1.12	0.39	5.15 ± 1.09	4.94 ± 0.96	0.12
Triglyceride (mmol/L)	1.56 (1.03, 2.19)	1.21 (0.93, 1.96)	0.16	1.52 (1.17, 1.99)	1.60 (1.10, 2.21)	0.51
HDL cholesterol (mmol/L)	1.16 ± 0.23	1.20 ± 0.21	0.40	1.21 ± 0.29	1.15 ± 0.25	0.08
LDL cholesterol (mmol/L)	3.38 ± 0.83	3.13 ± 0.90	0.18	3.26 ± 0.84	3.04 ± 0.69	0.04
Free fatty acid (μmol/L)	572 (461, 701)	544 (448, 640)	0.22	527 (449, 672)	493 (393, 598)	0.03
Fasting glucose (mmol/L)	5.0 (4.5, 5.6)	4.7 (4.4, 5.1)	0.04	4.9 (4.6, 5.7)	4.9 (4.4, 5.2)	0.18
Fasting insulin (uU/mL)	11.0 (7.9, 16.3)	11.8 (7.7, 18.4)	0.94	10.1 (7.8, 14.2)	9.9 (7.1, 13.6)	0.82
HOMA-IR	2.50 (1.86, 3.60)	2.51 (1.61, 4.06)	0.71	2.31 (1.64, 3.46)	2.20 (1.50, 3.19)	0.51
Uric acid (μmol/L)	422.3 ± 110.7	419.5 ± 95.8	0.90	427.7 ± 81.5	417.0 ± 108.8	0.38
Alanine aminotransferase (IU/L)	49.0 (31.0, 80.0)	40.0 (27.8, 71.3)	0.28	45.0 (27.3, 71.8)	35.0 (22.0, 56.0)	0.04
Aspartate aminotransferase (IU/L)	37.0 (26.0, 51.0)	27.0 (23.0, 46.3)	0.12	32.0 (24.3, 45.5)	28.0 (22.0, 39.0)	0.04
γ-glutamyl transpeptidase (IU/L)	48.0 (28.0, 70.0)	38.5 (22.0, 54.0)	0.16	43.0 (28.3, 65.8)	37.5 (25.0, 59.8)	0.16
Alkaline phosphatase (IU/L)	77.0 (69.0, 89.0)	75.5 (66.8, 84.3)	0.27	77.0 (66.0, 87.0)	76.0 (64.0, 86.0)	0.31
Total bilirubin (μmol/L)	13.5 (11.1, 17.0)	11.9 (9.9, 15.9)	0.16	12.3 (9.7, 16.8)	12.3 (9.7, 16.4)	0.59
Albumin (g/L)	45.6 ± 3.1	45.3 ± 3.3	0.66	45.7 ± 3.1	45.9 ± 3.0	0.67
Total bile acid (μmol/L)	2.6 (1.8, 3.8)	2.8 (2.1, 4.9)	0.37	2.6 (1.7, 3.9)	3.0 (1.8, 3.9)	0.43
Liver fat content (%)	16.1 (11.9, 25.0)	10.0 (7.5, 18.2)	0.001	14.1 (10.3, 22.2)	10.6 (7.5, 16.5)	<0.001
Liver stiffness measurement (kpa)	6.5 (5.5, 7.8)	6.1 (4.9, 7.2)	0.27	5.9 (5.0, 6.9)	5.8 (5.1, 6.8)	0.88
Moderate-vigorous physical activity ≥150 min/wk, n (%)	67 (73.6%)	15 (50.0%)	0.02	49 (61.3%)	120 (61.2%)	0.97
Reduced energy intake >500 kcal/d, n (%)	57 (62.6%)	16 (53.3%)	0.38	37 (46.3%)	76 (38.8%)	0.36
Lipid-lowering drug, n (%)	24 (26.4%)	12 (40.0%)	0.16	24 (30.0%)	69 (35.2%)	0.41
Uric acid-lowering drug, n (%)	7 (7.7%)	4 (13.3%)	0.57	10 (12.5%)	18 (9.2%)	0.41
ALT repsonse[a], n (%)	54 (79.4%)	8 (40.0%)	0.001	29 (52.7%)	36 (32.7%)	0.01
LSM response[a], n (%)	23 (52.3%)	6 (50.0%)	0.89	13 (52.0%)	21 (32.3%)	0.08

Continuous variables that are normally distributed are reported as mean ± standard deviation, while continuous variables that are not normally distributed are presented as median (interquartile range). Categorical data are expressed as frequency (percentage). Abbreviation: MASLD, metabolic dysfunction-associated steatotic liver disease; WLT, weight loss target; MRI-PDFF, magnetic resonance imaging proton density fat fraction; HDL cholesterol, high-density lipoprotein cholesterol; LDL cholesterol, low-density lipoprotein cholesterol; HOMA-IR, homeostasis model assessment of insulin resistance; ALT, alanine aminotransferase; LSM, liver stiffness measurement.

[a] ALT response status was evaluated in 253 MASLD patients with elevated ALT (>33 IU/L for men and >25 IU/L for women) at baseline, while LSM response status was analyzed in 146 MASLD patients with fibrosis stage ≥1 (LSM>6.4kpa) at baseline.

Table S3. Baseline characteristics of patients with MASLD stratified by ALT response status[a].

Characteristics	With WLT (n=88)			Without WLT (n=165)		
	ALT response (n=62)	ALT non-response (n=26)	*P*	ALT response (n=65)	ALT non-response (n=100)	*P*
Age (years)	38.3 ± 11.5	45.46 ± 14.25	0.02	39.4 ± 13.3	38.53 ± 13.05	0.68
Male, n (%)	45 (72.6%)	16 (61.5%)	0.31	54 (83.1%)	79 (79.00)	0.52
Smoking, n (%)	10 (16.1%)	2 (7.7%)	0.48	12 (18.8%)	13 (13.13)	0.33
Body weight (kg)	78.3 ± 13.3	78.2 ± 10.8	0.97	76.0 ± 10.4	73.6 ± 12.8	0.19
Body mass index (kg/m^2)	28.0 ± 3.6	28.1 ± 3.0	0.93	26.9 ± 3.1	26.1 ± 3.4	0.12
Waist circumference (cm)	92.1 ± 9.3	93.0 ± 7.8	0.64	90.8 ± 6.4	88.2 ± 8.1	0.03
Waist-to-hip ratio	0.90 ± 0.05	0.91 ± 0.05	0.67	0.90 ± 0.04	0.89 ± 0.05	0.09
Systolic blood pressure (mmHg)	130.2 ± 16.9	136.5 ± 14.8	0.11	133.3 ± 16.3	131.3 ± 16.6	0.46
Diastolic blood pressure(mmHg)	85.8 ± 12.8	90.0 ± 9.5	0.14	87.9 ± 11.3	84.4 ± 12.7	0.08
Total cholesterol (mmol/L)	5.55 ± 1.07	4.98 ± 1.19	0.03	5.41 ± 1.07	4.91 ± 0.92	0.002
Triglyceride (mmol/L)	1.71 (1.18, 2.28)	1.63 (1.08, 2.23)	0.61	1.76 (1.41, 2.41)	1.62 (1.15, 2.31)	0.12
HDL cholesterol (mmol/L)	1.14 ± 0.19	1.17 ± 0.30	0.51	1.16 ± 0.29	1.13 ± 0.26	0.47
LDL cholesterol (mmol/L)	3.62 ± 0.74	3.05 ± 0.97	0.004	3.40 ± 0.82	3.05 ± 0.64	0.003
Free fatty acid (μmol/L)	573 (476, 712)	587 (522, 737)	0.56	518 (397, 642)	501 (434, 597)	0.68
Fasting glucose (mmol/L)	5.0 (4.5, 5.6)	4.8 (4.4, 5.3)	0.44	5.0 (4.4, 6.1)	4.8 (4.5, 5.2)	0.21
Fasting insulin (uU/mL)	12.4 (9.6, 19.5)	11.2 (8.7, 19.3)	0.44	11.5 (8.3, 16.4)	9.37 (6.9, 13.2)	0.02
HOMA-IR	2.72 (2.13, 4.25)	2.55 (1.92, 4.05)	0.52	2.62 (1.81, 4.27)	2.03 (1.40, 3.00)	0.008
Uric acid (μmol/L)	439.1 ± 116.3	421.4 ± 77.4	0.48	459.8 ± 106.7	430.4 ± 106.4	0.09
Alanine aminotransferase (IU/L)	76.0 (52.3, 104.8)	39.5 (34.0, 48.3)	<0.001	74.0 (59.0, 125.0)	44.5 (36.0, 59.0)	<0.001
Aspartate aminotransferase (IU/L)	44.0 (37.0, 60.5)	27.5 (25.3, 41.5)	<0.001	47.0 (38.0, 62.0)	33.0 (25.8, 39.3)	<0.001
γ-glutamyl transpeptidase (IU/L)	54.0 (42.3, 90.3)	44.0 (31.5, 67.8)	0.14	56.0 (43.0, 102.0)	43.6 (32.8, 69.0)	0.004
Alkaline phosphatase (IU/L)	77.0 (68.3, 88.8)	80.0 (70.3, 90.8)	0.45	80.0 (69.0, 93.0)	75.0 (61.8, 85.3)	0.052
Total bilirubin (μmol/L)	13.7 (11.0, 17.0)	12.3 (9.9, 14.1)	0.08	13.3 (10.1, 17.9)	12.2 (9.6, 15.9)	0.12
Albumin (g/L)	46.0 ± 2.9	44.9 ± 3.8	0.13	46.5 ± 3.2	46.2 ± 3.1	0.68
Total bile acid (μmol/L)	2.7 (2.1, 4.3)	2.7 (2.1, 3.7)	0.50	3.8 (2.2, 3.9)	3.0 (1.8, 3.9)	0.28
Liver fat content (%)	19.7 (13.7, 26.2)	14.2 (9.5, 21.4)	0.02	17.7 (10.2, 25.0)	14.0 (9.6, 20.2)	0.09
Liver stiffness measurement (kpa)	6.3 (5.5, 7.8)	6.7 (5.6, 7.5)	0.93	6.1 (5.4, 7.1)	5.7 (5.0, 6.7)	0.07
Moderate-vigorous physical activity ≥150 min/wk, n (%)	35 (70.0%)	13 (65.0%)	0.68	24 (58.5%)	51 (71.8%)	0.15
Reduced energy intake >500 kcal/d, n (%)	35 (70.0%)	10 (47.6%)	0.07	11 (26.8%)	27 (38.0%)	0.23
Lipid-lowering drug, n (%)	15 (24.2%)	10 (38.5%)	0.18	18 (27.7%)	31 (31.0%)	0.65
Uric acid-lowering drug, n (%)	7 (11.3%)	2 (7.7%)	0.90	10 (15.4%)	10 (10.0%)	0.30
MRI-PDFF repsonse, n (%)	54 (87.1%)	14 (53.9%)	<0.001	29 (44.6%)	26 (26.0%)	0.01
LSM response[b], n (%)	13 (46.4%)	7 (50.0%)	0.83	7 (30.4%)	12 (41.4%)	0.42

Continuous variables that are normally distributed are reported as mean ± standard deviation, while continuous variables that are not normally distributed are presented as median (interquartile range). Categorical data are expressed as frequency (percentage). Abbreviation: MASLD, metabolic dysfunction-associated steatotic liver disease; WLT, weight loss target; ALT, alanine aminotransferase; HDL cholesterol, high-density lipoprotein cholesterol; LDL cholesterol, low-density lipoprotein cholesterol; HOMA-IR, homeostasis model assessment of insulin resistance; LSM, liver stiffness measurement.

[a] Comparison of baseline characteristics in 253 MASLD patients with elevated ALT at baseline.

[b] LSM response status was analyzed in 94 MASLD patients with both elevated ALT and fibrosis stage ≥1 at baseline.

Table S4. Baseline characteristics of patients with MASLD stratified by LSM response status[a].

Characteristics	With WLT (n=56)			Without WLT (n=90)		
	LSM response (n=29)	LSM non-response (n=27)	*P*	LSM response (n=34)	LSM non-response (n=56)	*P*
Age (years)	42.0 ± 11.0	46.2 ± 15.1	0.24	46.6 ± 12.6	47.3 ± 14.4	0.82
Male, n (%)	20 (69.0%)	17 (63.0%)	0.64	27 (79.4%)	41 (73.2%)	0.51
Smoking, n (%)	2 (6.9%)	2 (7.4%)	1.00	4 (11.8%)	6 (10.7%)	1.00
Body weight (kg)	79.0 ± 11.4	77.4 ± 14.1	0.64	73.1 ± 11.6	76.8 ± 14.7	0.22
Body mass index (kg/m^2)	28.4 ± 2.8	28.1 ± 3.9	0.77	26.5 ± 3.4	27.4 ± 3.9	0.25
Waist circumference (cm)	93.3 ± 8.1	93.6 ± 8.3	0.89	90.6 ± 6.6	91.6 ± 9.2	0.57
Waist-to-hip ratio	0.91 ± 0.04	0.92 ± 0.05	0.59	0.91 ± 0.04	0.91 ± 0.04	0.66
Systolic blood pressure (mmHg)	134.5 ± 16.3	135.7 ± 15.6	0.78	133.4 ± 14.6	136.4 ± 18.0	0.42
Diastolic blood pressure(mmHg)	89.4 ± 13.0	88.5 ± 10.1	0.77	87.8 ± 11.2	84.7 ± 10.5	0.19
Total cholesterol (mmol/L)	5.06 ± 0.98	5.38 ± 1.46	0.34	5.08 ± 0.88	5.01 ± 0.98	0.74
Triglyceride (mmol/L)	1.53 (0.98, 1.87)	1.56 (0.86, 2.77)	0.65	1.52 (1.15, 2.09)	1.55 (0.98, 2.38)	0.86
HDL cholesterol (mmol/L)	1.10 ± 0.21	1.15 ± 0.17	0.35	1.15 ± 0.27	1.19 ± 0.29	0.48
LDL cholesterol (mmol/L)	3.32 ± 0.77	3.37 ± 0.94	0.83	3.25 ± 0.70	3.09 ± 0.70	0.28
Free fatty acid (μmol/L)	520 (429, 690)	618 (563, 762)	0.007	546 (403, 757)	520 (434, 631)	0.54
Fasting glucose (mmol/L)	4.7 (4.5, 5.3)	5.7 (4.5, 6.8)	0.07	5.2 (4.9, 6.1)	5.2 (4.7, 6.7)	0.96
Fasting insulin (uU/mL)	11.7 (7.7, 19.7)	10.8 (8.5, 20.7)	0.85	9.8 (8.1, 14.3)	11.2 (7.3, 16.4)	0.44
HOMA-IR	2.44 (1.52, 4.74)	2.65 (2.06, 5.36)	0.35	2.44 (1.70, 3.45)	2.57 (1.70, 4.37)	0.47
Uric acid (μmol/L)	395.6 ± 98.2	427.7 ± 106.2	0.25	433.1 ± 97.6	428.5 ± 105.5	0.84
Alanine aminotransferase (IU/L)	40.0 (32.0, 97.0)	48.0 (29.0, 84.0)	0.81	35.0 (20.8, 56.0)	34.5 (27.0, 71.5)	0.34
Aspartate aminotransferase (IU/L)	42.0 (25.0, 65.5)	37.0 (28.0, 59.0)	0.74	32.0 (21.0, 42.5)	30.0 (24.0, 43.8)	0.53
γ-glutamyl transpeptidase (IU/L)	50.0 (26.5, 87.0)	51.0 (34.0, 76.0)	0.61	35.5 (26.8, 48.8)	34.0 (24.0, 60.0)	0.99
Alkaline phosphatase (IU/L)	78.0 (69.0, 85.5)	85.0 (71.0, 92.0)	0.19	73.5 (61.0, 80.0)	78.0 (68.3, 88.5)	0.10
Total bilirubin (μmol/L)	13.1 (10.9, 16.5)	13.5 (11.3, 17.2)	0.56	13.9 (9.8, 17.7)	12.5 (10.0, 16.8)	0.58
Albumin (g/L)	44.7 ± 3.2	45.2 ± 3.5	0.60	45.3 ± 2.6	45.7 ± 2.8	0.53
Total bile acid (μmol/L)	2.7 (2.0, 3.7)	3.0 (1.9, 4.1)	0.84	3.3 (2.1, 3.9)	3.4 (2.0, 4.9)	0.59
Liver fat content (%)	12.4 (8.1, 21.2)	18.2 (13.7, 27.1)	0.02	13.4 (8.7, 23.3)	11.2 (7.6, 19.9)	0.36
Liver stiffness measurement (kpa)	7.8 (7.1, 9.4)	7.5 (6.6, 9.4)	0.70	7.5 (6.7, 8.8)	7.6 (6.8, 8.5)	0.85
Moderate-vigorous physical activity ≥150 min/wk, n (%)	23 (79.3%)	19 (70.3%)	0.39	20 (58.8%)	35 (62.5%)	0.79
Reduced energy intake ≥500 kcal/d, n (%)	19 (65.5%)	12 (44.4%)	0.15	14 (41.2%)	29 (51.8%)	0.44
Lipid-lowering drug, n (%)	9 (31.0%)	7 (25.9%)	0.67	11 (32.4%)	22 (39.3%)	0.51
Uric acid lowering drug, n (%)	3 (10.3%)	2 (7.4%)	1.00	6 (17.6%)	6 (10.7%)	0.54
MRI-PDFF repsonse, n (%)	23 (79.3%)	21 (77.8%)	0.89	13 (38.2%)	12 (21.4%)	0.08
ALT response[b], n (%)	13 (65.0%)	15 (68.2%)	0.83	7 (36.8%)	16 (48.5%)	0.42

Continuous variables that are normally distributed are reported as mean ± standard deviation, while continuous variables that are not normally distributed are presented as median (interquartile range). Categorical data are expressed as frequency (percentage). Abbreviation: MASLD, metabolic dysfunction-associated steatotic liver disease; WLT, weight loss target; LSM, liver stiffness measurement; HDL cholesterol, high-density lipoprotein cholesterol; LDL cholesterol, low-density lipoprotein cholesterol; HOMA-IR, homeostasis model assessment of insulin resistance; MRI-PDFF, magnetic resonance imaging proton density fat fraction; ALT, alanine aminotransferase.

[a] Comparison of baseline characteristics in 146 MASLD patients with fibrosis stage ≥1 at baseline.

[b] ALT response status was evaluated in 94 MASLD patients with both abnormal ALT and fibrosis stage ≥1 at baseline.

Table S5. Factors associated with treatment non-response to hepatic steatosis, injury, or fibrosis in patients with MASLD who achieved weight loss targets.

Predictors	MRI-PDFF non-response (n=121)		ALT non-response (n=88)		LSM non-response (n=56)	
	Univariable OR (95%CI)	Multivariable OR (95%CI)	Univariable OR (95%CI)	Multivariable OR (95%CI)	Univariable OR (95%CI)	Multivariable OR (95%CI)
Age (years)	1.01 (0.98-1.04)	1.00 (0.94-1.05)	1.05 (1.01-1.09)*	1.03 (0.94-1.12)	1.03 (0.98-1.07)	1.04 (0.98-1.10)
Male	0.89 (0.38-2.11)	0.73 (0.19-2.82)	0.60 (0.23-1.59)	2.56 (0.31-21.50)	0.77 (0.25-2.32)	1.31 (0.26-6.73)
Hypertension	1.19 (0.49-2.86)		2.69 (1.02-7.05)*	4.38 (0.62-30.91)	1.12 (0.37-3.34)	
Smoking	0.26 (0.03-2.10)		0.43 (0.09-2.13)		1.13 (0.15-8.61)	
MVPA ≥150 min/wk	0.34 (0.14-0.86)*	**0.29 (0.09-0.94)***	0.80 (0.26-2.39)		0.59 (0.17-2.00)	
Reduced energy intake ≥500 kcal/d	0.67 (0.28-1.64)		0.39 (0.14-1.11)		0.45 (0.15-1.34)	
Lipid-lowering drug	1.86 (0.78-4.43)	0.73 (0.19-2.74)	1.96 (0.73-5.22)	0.84 (0.15-4.63)	0.78 (0.24-2.50)	0.73 (0.17-3.21)
Uric acid-lowering drug	1.85 (0.50-6.81)	5.25 (0.83-33.16)	0.66 (0.13-3.39)	16.29 (0.65-41.92)	0.69 (0.11-4.51)	0.34 (0.03-4.55)
**Indicators at baseline**						
Body mass index (kg/m^2)	1.10 (0.97-1.24)		1.01 (0.88-1.15)		0.98 (0.83-1.14)	
Waist circumference (cm)	1.02 (0.97-1.07)		1.01 (0.96-1.07)		1.00 (0.94-1.07)	
Triglyceride (mmol/L)	0.72 (0.43-1.19)		0.75 (0.46-1.25)		1.36 (0.84-2.21)	
HDL cholesterol (mmol/L)	2.19 (0.36-13.48)		1.98 (0.26-15.06)		3.86 (0.23-64.88)	
LDL cholesterol (mmol/L)	0.71 (0.43-1.17)		0.42 (0.23-0.79)**	0.33 (0.11-1.03)	1.07 (0.58-2.00)	
FFA per 100 μmol/L increase	0.83 (0.65-1.06)		1.07 (0.82-1.38)		1.69 (1.14-2.50)**	**1.53 (1.01-2.32)***
Fasting glucose (mmol/L)	0.67 (0.41-1.11)		0.90 (0.58-1.39)		1.53 (0.96-2.44)	
Fasting insulin (uU/mL)	1.00 (0.95-1.06)		0.99 (0.93-1.05)		1.00 (0.94-1.07)	
HOMA-IR	0.97 (0.80-1.19)		0.93 (0.74-1.16)		1.06 (0.86-1.32)	
UA per 100 μmol/L increase	0.98 (0.66-1.44)		0.85 (0.54-1.33)		1.37 (0.81-2.34)	
ALT per 10 IU/L increase	0.97 (0.87-1.08)		0.49 (0.34-0.70)***	**0.43 (0.25-0.74)****	1.03 (0.92-1.16)	
GGT per 10 IU/L increase	0.95 (0.85-1.06)		0.95 (0.85-1.06)		0.98 (0.91-1.07)	
Total bilirubin (μmol/L)	0.95 (0.87-1.03)		0.91 (0.81-1.01)		1.04 (0.94-1.16)	
Albumin (g/L)	0.97 (0.85-1.11)		0.89 (0.77-1.04)		1.05 (0.89-1.23)	
Total bile acid (μmol/L)	0.99 (0.92-1.07)		0.95 (0.80-1.13)		0.96 (0.85-1.08)	
LFC per 5% increase	0.71 (0.53-0.96)*	0.81 (0.52-1.27)	0.70 (0.52-0.96)*	1.70 (0.71-4.08)	1.41 (1.01-1.96)*	1.41 (0.90-2.21)
LSM (kpa)	1.01 (0.92-1.11)		1.04 (0.94-1.16)		1.03 (0.93-1.15)	
**Changes of indicators**						
△Waist circumference (cm)	0.92 (0.84-1.02)		0.95 (0.86-1.05)		1.04 (0.90-1.19)	
△Triglyceride (mmol/L)	0.75 (0.45-1.25)		0.86 (0.52-1.40)		1.23 (0.69-2.18)	
△HDL cholesterol (mmol/L)	2.03 (0.26-15.79)		0.72 (0.10-5.28)		0.19 (0.01-2.50)	
△LDL cholesterol (mmol/L)	0.73 (0.41-1.31)		0.71 (0.37-1.35)		1.18 (0.57-2.43)	
△FFA per 100 μmol/L increase	0.84 (0.69-1.02)		0.90 (0.72-1.12)		1.06 (0.82-1.37)	
△Fasting glucose (mmol/L)	0.53 (0.28-0.98)*	0.51 (0.21-1.23)	0.86 (0.49-1.51)		1.21 (0.68-2.16)	
△Fasting insulin (uU/mL)	1.00 (0.93-1.06)		0.99 (0.92-1.06)		0.96 (0.89-1.04)	
△HOMA-IR	0.95 (0.75-1.21)		0.94 (0.74-1.20)		0.93 (0.73-1.19)	
△UA per 100 μmol/L increase	1.19 (0.80-1.78)		0.86 (0.57-1.31)		1.15 (0.70-1.88)	
△ALT per 10 IU/L increase	0.86 (0.74-0.99)*	0.98 (0.96-1.01)	-	-	1.02 (0.89-1.17)	
△GGT per 10 IU/L increase	0.91 (0.78-1.06)		0.79 (0.65-0.96)*	0.99 (0.95-1.02)	0.94 (0.82-1.07)	
△Total bilirubin (μmol/L)	1.01 (0.91-1.11)		0.92 (0.82-1.04)		1.04 (0.91-1.20)	
△Albumin (g/L)	1.08 (0.94-1.24)		1.01 (0.85-1.20)		0.94 (0.77-1.14)	
△Total bile acid (μmol/L)	1.02 (0.91-1.15)		1.15 (0.95-1.40)		1.03 (0.92-1.14)	
△LFC per 5% increase	-	-	0.48 (0.31-0.75)**	**0.23 (0.08-0.62)****	1.10 (0.73-1.65)	
△LSM (kpa)	1.07 (0.92-1.23)		1.06 (0.88-1.28)		-	-

Abbreviation: MASLD, metabolic dysfunction-associated steatotic liver disease; MVPA, Moderate-vigorous physical activity; HDL cholesterol, high-density lipoprotein cholesterol; LDL cholesterol, low-density lipoprotein cholesterol; FFA, free fatty acid; HOMA-IR, homeostasis model assessment of insulin resistance; UA, uric acid; ALT, alanine aminotransferase; GGT, γ-glutamyl transpeptidase; LFC, liver fat content; LSM, liver stiffness measurement; △, change in indicators i.e. baseline minus 48-week measurements.
Age, sex, and variables with P <0.05 in univariable analysis were included in multivariable logistic models.
$^{*}P$<0.05, $^{**}P$<0.01, $^{***}P$<0.001.
Bold type indicates the OR (95% CI) of the clinical indicators with statistically significant results ($^{*}P$<0.05).

Table S6. Factors associated with treatment response to hepatic steatosis, injury, or fibrosis in patients with MASLD who did not achieve weight loss targets.

Predictors	MRI-PDFF response (n=276)		ALT response (n=165)		LSM response (n=90)	
	Univariable OR (95%CI)	Multivariable OR (95%CI)	Univariable OR (95%CI)	Multivariable OR (95%CI)	Univariable OR (95%CI)	Multivariable OR (95%CI)
Age (years)	1.01 (0.99-1.03)	1.01 (0.99-1.04)	1.01 (0.98-1.03)	1.04 (0.98-1.11)	1.00 (0.97-1.03)	1.00 (0.96-1.04)
Male	0.66 (0.37-1.16)	0.70 (0.33-1.47)	1.30 (0.58-2.93)	3.00 (0.58-15.53)	1.41 (0.51-3.91)	1.28 (0.37-4.36)
Hypertension	1.62 (0.90-2.92)		0.94 (0.43-2.04)		0.52 (0.20-1.31)	
Smoking	0.64 (0.27-1.56)		1.53 (0.65-3.60)		1.09 (0.28-4.18)	
MVPA ≥150 min/wk	1.01 (0.53-1.94)		0.55 (0.25-1.24)		0.87 (0.30-2.51)	
Reduced energy intake ≥500 kcal/d	1.34 (0.71-2.54)		0.60 (0.26-1.39)		0.66 (0.23-1.88)	
Lipid-lowering drug	0.79 (0.45-1.38)	0.58 (0.29-1.17)	0.85 (0.43-1.70)	1.90 (0.51-7.00)	0.74 (0.30-1.81)	0.61 (0.22-1.68)
Uric acid-lowering drug	1.41 (0.62-3.21)	1.08 (0.42-2.77)	1.64 (0.64-4.18)	1.77 (0.39-8.17)	1.79 (0.53-6.06)	1.49 (0.39-5.65)
**Indicators at baseline**						
Body mass index (kg/m^2)	1.01 (0.94-1.09)		1.08 (0.98-1.19)		0.93 (0.83-1.05)	
Waist circumference (cm)	1.01 (0.97-1.04)		1.05 (1.01-1.09)*	1.02 (0.95-1.10)	0.99 (0.94-1.04)	
Triglyceride (mmol/L)	0.86 (0.68-1.10)		1.11 (0.89-1.39)		0.87 (0.58-1.31)	
HDL cholesterol (mmol/L)	2.38 (0.91-6.23)		1.52 (0.48-4.79)		0.57 (0.12-2.72)	
LDL cholesterol (mmol/L)	1.49 (1.05-2.13)*	0.94 (0.58-1.53)	1.95 (1.24-3.07)**	0.60 (0.23-1.56)	1.41 (0.76-2.61)	
FFA per 100 μmol/L increase	1.16 (1.00-1.33)*	1.02 (0.87-1.20)	1.03 (0.99-1.07)		1.15 (0.90-1.47)	
Fasting glucose (mmol/L)	1.19 (0.93-1.53)		1.50 (1.09-2.05)*	1.10 (0.57-2.12)	0.86 (0.62-1.21)	
Fasting insulin (uU/mL)	1.00 (0.96-1.04)		1.03 (0.99-1.07)		0.98 (0.92-1.04)	
HOMA-IR	1.02 (0.89-1.16)		1.21 (1.02-1.42)*	0.93 (0.66-1.31)	0.93 (0.77-1.13)	
UA per 100 μmol/L increase	1.11 (0.86-1.43)		1.30 (0.96-1.76)		1.05 (0.69-1.59)	
ALT per 10 IU/L increase	1.04 (0.98-1.10)		1.59 (1.35-1.87)***	**2.39 (1.70-3.35)*****	0.90 (0.79-1.01)	
GGT per 10 IU/L increase	1.03 (0.99-1.08)		1.05 (1.01-1.11)*	**0.86 (0.77-0.96)****	0.97 (0.87-1.09)	
Total bilirubin (μmol/L)	1.01 (0.97-1.05)		1.04 (0.99-1.08)		1.02 (0.93-1.12)	
Albumin (g/L)	0.98 (0.90-1.07)		1.02 (0.92-1.13)		0.95 (0.81-1.11)	
Total bile acid (μmol/L)	0.99 (0.94-1.04)		0.98 (0.92-1.04)		0.95 (0.83-1.08)	
LFC per 5% increase	1.31 (1.11-1.54)**	**1.23 (1.01-1.49)***	1.22 (1.01-1.46)*	0.64 (0.41-1.03)	1.11 (0.87-1.42)	
LSM (kpa)	1.00 (0.91-1.10)		1.05 (0.94-1.17)		0.89 (0.74-1.07)	
**Changes of indicators**						
△Waist circumference (cm)	1.17 (1.08-1.26)***	**1.13 (1.05-1.23)****	1.04 (0.97-1.12)		1.06 (0.94-1.19)	
△Triglyceride (mmol/L)	0.97 (0.78-1.20)		1.17 (0.91-1.51)		0.71 (0.42-1.19)	
△HDL cholesterol (mmol/L)	0.65 (0.19-2.19)		1.23 (0.28-5.38)		0.63 (0.07-6.06)	
△LDL cholesterol (mmol/L)	1.70 (1.21-2.38)**	**1.68 (1.05-2.70)***	1.93 (1.26-2.97)**	2.48 (0.99-6.16)	1.00 (0.61-1.65)	
△FFA per 100 μmol/L increase	1.00 (0.88-1.13)		1.10 (0.93-1.29)		0.99 (0.80-1.22)	
△Fasting glucose (mmol/L)	1.04 (0.79-1.37)		1.05 (0.77-1.42)		0.70 (0.45-1.10)	
△Fasting insulin (uU/mL)	1.02 (0.98-1.07)		1.04 (0.98-1.09)		0.94 (0.86-1.03)	
△HOMA-IR	1.07 (0.90-1.29)		1.18 (0.96-1.45)		0.79 (0.59-1.06)	
△UA per 100μmol/L increase	1.27 (0.99-1.65)		1.34 (1.00-1.80)		0.88 (0.61-1.26)	
△ALT per 10 IU/L increase	1.07 (1.01-1.13)*	1.01 (0.93-1.09)	-	-	0.89 (0.78-1.01)	
△GGT per 10 IU/L increase	1.19 (1.08-1.30)***	1.12 (0.99-1.25)	1.37 (1.19-1.58)***	**1.44 (1.19-1.74)*****	0.95 (0.84-1.08)	
△Total bilirubin (μmol/L)	0.98 (0.93-1.04)		1.04 (0.98-1.11)		0.96 (0.84-1.09)	
△Albumin (g/L)	1.01 (0.92-1.12)		1.04 (0.93-1.16)		0.97 (0.83-1.14)	
△Total bile acid (μmol/L)	1.00 (0.95-1.05)		0.96 (0.87-1.05)		1.00 (0.95-1.06)	
△LFC per 5% increase	-	-	1.72 (1.27-2.32)***	**1.97 (1.03-3.75)***	1.58 (1.05-2.39)*	**1.57 (1.03-2.39)***
△LSM (kpa)	1.20 (0.97-1.48)		1.18 (0.92-1.50)		-	-

Abbreviation: MASLD, metabolic dysfunction-associated steatotic liver disease; MVPA, Moderate-vigorous physical activity; HDL cholesterol, high-density lipoprotein cholesterol; LDL cholesterol, low-density lipoprotein cholesterol; FFA, free fatty acid; HOMA-IR, homeostasis model assessment of insulin resistance; UA, uric acid; ALT, alanine aminotransferase; GGT, γ-glutamyl transpeptidase; LFC, liver fat content; LSM, liver stiffness measurement; △, change in indicators i.e. baseline minus 48-week measurements.
Age, sex, and variables with P <0.05 in univariable analysis were included in multivariable logistic models.
$^{*}P$<0.05, $^{**}P$<0.01, $^{***}P$<0.001.
Bold type indicates the OR (95% CI) of the clinical indicators with statistically significant results ($^{*}P$<0.05).

Supplementary File S1

中山大學 附属第一医院
The First Affiliated Hospital,Sun Yat-sen University

Time of enrollment (DD/MM/YY)		Identification Number				

Questionnaire on personal lifestyle for the participants in a prospective observational study of fatty liver disease

The institutional ethics approved code: [2014]112.

Part 1. Demographic characteristics

Name		Gender	Male/Female
Age	_____years	Date of Birth	____/____/____
Address	City ____________; Sub-city ___________; Woreda _______________		
Phone number			
Education	☐Illiteracy<br>☐Primary school<br>☐Middle school<br>☐Junior college<br>☐University and above		
Occupation	☐Employed whether you are manual worker or not: Yes/No<br>☐Unemployed ____________		

Part 2. Medical history

History of present illness	☐Diabetes mellitus<br>☐Hypertension<br>☐Chronic heart disease<br>☐Cerebrovascular disease<br>☐Viral hepatitis<br>☐Others__________
Previous medical history	
Smoking	☐Never or past<br>☐Current (average <10 cigarettes per day)<br>☐Current (average 10–20 cigarettes per day)<br>☐Current (average >20 cigarettes per day)<br>*Current: smoke in last one year
Drinking	☐Never or past<br>☐Current, alcohol consumption:__________g per week<br>*Current: drink in last one year

Part 3. Physical activity

Next, I am going to ask you about the time you spend doing different types of physical activity in a typical week. Please answer these questions even if you do not consider yourself to be a physically active person. Think first about the time you spend doing work. Think of work as the things that you have to do such as paid or unpaid work, study/training, household chores, harvesting food/crops, fishing or hunting for food, seeking employment. In answering the following questions 'vigorous-intensity activities' are activities that require hard physical effort and cause large increases in breathing or heart rate, 'moderate-intensity activities' are activities that require moderate physical effort and cause small increases in breathing or heart rate.

Question	Response	Code
**Work**		
Does your work involve vigorous-intensity activity that causes large increases in breathing or heart rate like [carrying or lifting heavy loads, digging or construction work] for at least 30 minutes? 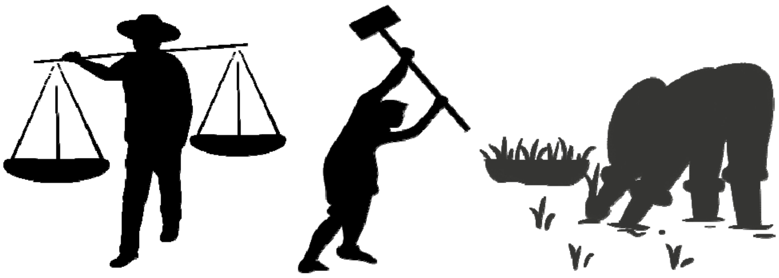	Yes 1<br><br>No 2<br>(If No, go to P4)	P1
In a typical week, on how many days do you do vigorous-intensity activities as part of your work?	Number of days____	P2
How much time do you spend doing vigorous-intensity activities at work on a typical day?	_____Hours:_____minutes	P3
Does your work involve moderate-intensity activity, that causes small increases in breathing or heart rate such as brisk walking [or carrying light loads] for at least 30 minutes? 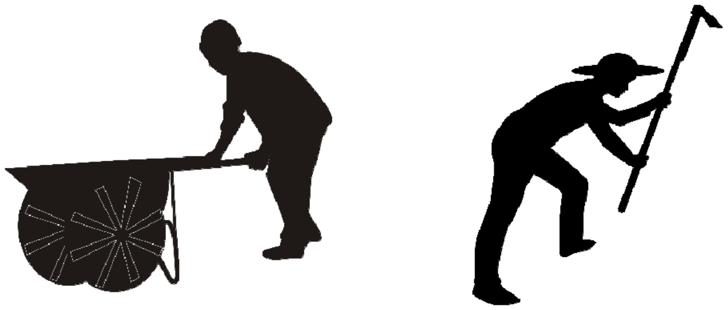	Yes 1<br><br>No 2<br>(If No, go to P 7)	P4
In a typical week, on how many days do you do moderate-intensity activities as part of your work?	Number of days _____	P5
How much time do you spend doing moderate-intensity activities at work on a typical day?	_____Hours_____minutes	P6
**Travel to and from places**		

The next questions exclude the physical activities at work that you have already mentioned. Now I would like to ask you about the usual way you travel to and from places. For example: to work, for shopping, to market, to place of worship.		
Do you walk or use a bicycle (pedal cycle) for at least 30 minutes to get to and from places?	Yes 1<br>No 2<br>(If No, go to P10)	P7
In a typical week, on how many days do you walk or bicycle for at least 30 minutes to get to and from places?	Number of days _____	P8
How much time do you spend walking or bicycling for travel on a typical day?	_____Hours_____minutes	P9
**Recreational activities**		
The next questions exclude the work and transport activities that you have already mentioned. Now I would like to ask you about sports, fitness and recreational activities (leisure)		
Do you do any vigorous-intensity sports, fitness or recreational (leisure) activities that cause large increases in breathing or heart rate like [running or football] for at least 30 minutes?<br>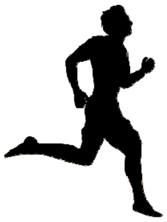 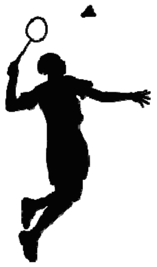	Yes 1<br><br>No 2<br>(If No, go to P13)	P10
In a typical week, on how many days do you do vigorous-intensity sports, fitness or recreational (leisure) activities?	Number of days _____	P11
How much time do you spend doing vigorous-intensity sports, fitness or recreational activities on a typical day?	_____Hours_____minutes	P12
Do you do any moderate-intensity sports, fitness or recreational (leisure) activities that cause a small increase in breathing or heart rate such as brisk walking, [cycling, swimming, volleyball] for at least 30 minutes?<br>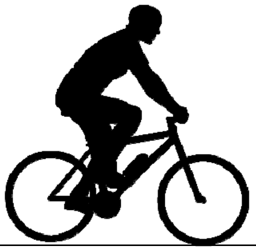 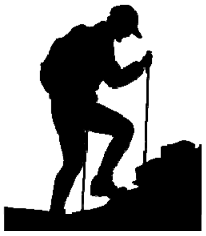	Yes 1<br><br>No 2<br>(If No, go to P16)	P13
In a typical week, on how many days do you do moderate-intensity sports, fitness or recreational (leisure) activities?	Number of days _____	P14
How much time do you spend doing moderate-intensity sports, fitness or recreational (leisure) activities on a typical day?	_____Hours_____minutes	P15

Part 4. Dietary habits

This form asks about your usual food intake during the recent month.

In the "how often did you eat these food" part, tick in the box to show how often, on average, you ate the following food. In the "amount" part, tick in the box to choose your usual serving size as small (S), median (M) or large (L).

◆ A small serving is about one-half (1/2) the medium serving size or less.

◆ A large serving is about one-and-a-half (3/2) times the medium serving size or more.

1. Cereals

	How often did you eat these foods?									→	Amount		
	Never or less than once per month	1 per month	2-3 per month	1 per week	2 per week	3-4 per week	5-6 per week	1 per day	2+ per day	Medium Serving size	S	M	L
Noodles										1 bowel			
Steamed roll										1 bun			
Steamed bread										1 bun			
Dumpling										1 dish			
Rice										1 bowel			
Rice flour										1 bowel			
Bread										2 slices			
Oat										1 cup			
Other________										1			

2. Fruits

	How often did you eat these foods?									→	Amount		
	Never or less than once per month	1 per month	2-3 per month	1 per week	2 per week	3-4 per week	5-6 per week	1 per day	2+ per day	Medium Serving size	S	M	L
Apple										1 medium			
Banana										1 medium			
Orange										1 medium			
Pear										1 medium			
Peach										1 medium			
Chinese gooseberry										1 medium			
Watermelon										1 medium slice			
Pineapple										1 medium			
Mango										1 medium			
Pomegranate										1 medium			
Other________										1 medium			

3. Vegetables

	How often did you eat these foods?	→	Amount

	Never or less than once per month	1 per month	2-3 per month	1 per week	2 per week	3-4 per week	5-6 per week	1 per day	2+ per day	Medium Serving size	S	M	L
Potato										1/2 cup			
Radish										1/2 cup			
Carrot										1/2 cup			
Brinjaul										1/2 cup			
Cauliflower										1/2 cup			
Legume										1/2 cup			
Capsicum										1/2 cup			
Tomato										1/2 cup			
Wax gourd										1/2 cup			
Pumpkin										1/2 cup			
Towel gourd										1/2 cup			
Celery cabbage										1/2 cup			
Greengrocery										1/2 cup			
Lettuce										1/2 cup			
Cole										1/2 cup			
Chinese kale										1/2 cup			
Celery										1/2 cup			
Asparagus										1/2 cup			
Green onion										1/2 cup			
Leek										1/2 cup			
Bean sprout										1/2 cup			
Other________										1/2 cup			
**4. Legumes**													
	How often did you eat these foods?									→	Amount		
	Never or less than once per month	1 per month	2-3 per month	1 per week	2 per week	3-4 per week	5-6 per week	1 per day	2+ per day	Medium Serving size	S	M	L
Soybean milk										1/2 cup			
Bean curd										1/2 cup			
Dried bean curd										1/2 cup			
Preserved bean curd										1/2 cup			
Others________										1/2 cup			
**5. Red meat**													
	How often did you eat these foods?									→	Amount		
	Never or less than once per month	1 per month	2-3 per month	1 per week	2 per week	3-4 per week	5-6 per week	1 per day	2+ per day	Medium Serving size	S	M	L
Pork										4 slices			
Lamb										4 slices			
Beef										4 slices			

Animal blood										3 pieces			
Animal liver										1 piece			
Others________													
**6. Poultry**													
	How often did you eat these foods?									→	Amount		
	Never or less than once per month	1 per month	2-3 per month	1 per week	2 per week	3-4 per week	5-6 per week	1 per day	2+ per day	Medium Serving size	S	M	L
Chicken										4 slices			
Duck										4 slices			
Goose										4 slices			
Others________													
**7. Fish**													
	How often did you eat these foods?									→	Amount		
	Never or less than once per month	1 per month	2-3 per month	1 per week	2 per week	3-4 per week	5-6 per week	1 per day	2+ per day	Medium Serving size	S	M	L
Marine fish										1 dish			
Fresh fish										1 dish			
Shrimp										1 dish			
Others________										1 dish			
**8. Eggs**													
	How often did you eat these foods?									→	Amount		
	Never or less than once per month	1 per month	2-3 per month	1 per week	2 per week	3-4 per week	5-6 per week	1 per day	2+ per day	Medium Serving size	S	M	L
Chicken eggs										1 egg			
Duck eggs										1 egg			
Salt eggs										1 egg			
Preserved eggs										1 egg			
Quail eggs										1 egg			
Others________										1 egg			
**9. Dairy**													
	How often did you eat these foods?									→	Amount		
	Never or less than once per month	1 per month	2-3 per month	1 per week	2 per week	3-4 per week	5-6 per week	1 per day	2+ per day	Medium Serving size	S	M	L
Cow’s milk										1 cup			
Goats’ milk										1 cup			
Yogurt										1 scoop			
Others________										1 cup			
**10. Food oil**													
	How often did you eat these foods?									→	Amount		

	Never or less than once per month	1 per month	2-3 per month	1 per week	2 per week	3-4 per week	5-6 per week	1 per day	2+ per day	Medium Serving size	S	M	L
Bean oil										1 spoon			
Rapeseed oil										1 spoon			
Salad oil										1 spoon			
Peanut oil										1 spoon			
Oliver oil										1 spoon			
Sesame oil										1 spoon			
Others________										1 spoon			
**11. Candy**													
	How often did you eat these foods?									→	Amount		
	Never or less than once per month	1 per month	2-3 per month	1 per week	2 per week	3-4 per week	5-6 per week	1 per day	2+ per day	Medium Serving size	S	M	L
Chocolate										1 regular bar or 2 pieces			
Cake										1 piece of cake			
Toffee										2 pieces			
Fruit confection										1 regular bar or 2 pieces			
Biscuit										4 pieces			
Others________													
**12. Beverage**													
	How often did you eat these foods?									→	Amount		
	Never or less than once per month	1 per month	2-3 per month	1 per week	2 per week	3-4 per week	5-6 per week	1 per day	2+ per day	Medium Serving size	S	M	L
Coffee										1 cup			
Black tea										1 cup			
Green tea										1 cup			
Red tea										1 cup			
Fruit juice										1 cup			
Diet soft drinks										1 cup			
Beer										12 once can or bottle			
Wine										1 medium glass (6 oz)			
